# Adiposity and the first-onset of diagnosed mental illnesses: a population-based cohort study of 10 million UK adults

**DOI:** 10.1186/s12916-025-04514-z

**Published:** 2025-11-29

**Authors:** Xue Dong, Paul Aveyard, Xiaochen Yang, Mika Kivimaki, Shanquan Chen, Joseph Firth, Cynthia Wright Drakesmith, Min Gao

**Affiliations:** 1 Nuffield Department of Primary Care Health Sciences, University of Oxford, Radcliffe Primary Care Building, Radcliffe Observatory Quarter, Woodstock Road, Oxford, OX2 6GG UK; 2 Center for Clinical and Epidemiologic Research, Beijing Anzhen Hospital, Capital Medical University, Beijing Institute of Heart, Lung and Blood Vessel Diseases, Beijing, People’s Republic of China; 3 NIHR Oxford Health Biomedical Research Centre, Warneford Hospital, Oxford, UK; 4 Department of Social Medicine and Health Education, School of Public Health, Peking University, Beijing, China; 5 University of Helsinki, Helsinki, Finland; 6 Brain Sciences, University College London, London, UK; 7 School of Public Health, The University of Hong Kong, Hong Kong, China; 8 Division of Psychology and Mental Health, University of Manchester, Manchester Academic Health Science Centre, Manchester, UK

**Keywords:** Body mass index, Depression, Anxiety, Serious mental illness, Eating disorder, Longitudinal

## Abstract

**Background:**

Whether both high and low BMI are risk factors for mental illnesses is unclear, especially serious mental illnesses. Experimental evidence suggests that metabolic pathways might mediate the association, but this is uncertain. To examine associations between BMI and the first-onset of diagnosed mental illnesses with assessment for mediating effects of cardiometabolic disorders.

**Methods:**

Using the UK Clinical Practice Research Datalink (2000–2022), individuals ≥ 18 years with a recorded BMI assessment were included. Diagnosed mental illnesses were ascertained via clinical diagnoses, referrals to mental health services, or psychotropic prescriptions. Restricted cubic splines and Cox proportional hazard models estimated associations between BMI and incident mental illnesses. Mediation analyses assessed the mediating role of cardiometabolic disorders and biomarkers.

**Results:**

The study population included 10,465,562 adults (mean BMI 26.8 kg/m^2^, standard deviation 5.5). Compared to those with a healthy BMI (18.5–25.0 kg/m^2^), individuals with severe obesity (≥ 40 kg/m^2^) had higher risks for depression (HR 1.32; 95% CI 1.31–1.33), anxiety (1.12; 1.10–1.13), bulimia nervosa (1.38; 1.20–1.58), other unspecified eating disorders (OUD) (2.04; 1.91–2.17), bipolar disorder (1.44; 1.36–1.54), schizophrenia (2.02; 1.91–2.15) and other psychoses (1.40; 1.28–1.53), but a lower risk for anorexia nervosa (0.45; 0.42–0.49). Individuals with underweight (< 18.5 kg/m^2^) were at increased risks of depression (1.28; 1.27–1.29), anxiety (1.26; 1.24–1.27), anorexia nervosa (3.88; 3.77–4.01), bulimia nervosa (1.52; 1.37–1.68), OUD (4.50; 4.33–4.67), schizophrenia (1.46; 1.35–1.56), and other psychoses (1.22; 1.10–1.35), with no association with bipolar disorder. Associations were stronger in women, younger adults, and Asian populations. Cardiometabolic diseases did not mediate associations, but LDL, triglycerides, HbA1c demonstrated partial mediation (indirect effect: 0.2%-15%).

**Conclusions:**

Individuals with severe obesity and underweight had increased risk of common and serious mental illnesses, especially in women, younger individuals, and Asian populations. Associations were not mediated by cardiometabolic diseases but partially by biomarkers.

**Supplementary Information:**

The online version contains supplementary material available at 10.1186/s12916-025-04514-z.

## Background

One in eight people worldwide is diagnosed with a mental illness, ranging from common conditions like depression and anxiety to more serious mental illnesses (SMI), such as schizophrenia, bipolar disorder, and psychoses [1]. Mental illnesses are leading causes of disability-adjusted life years (DALYs) globally, with age-standardised DALYs for depression and anxiety increasing by 16.4% and 16.7%, respectively, between 2010 and 2021 [2]. Identifying modifiable risk factors, such as adiposity, is critical to reducing this burden.

Reviews suggest that 20%−60% of individuals with obesity experience a mental illness [3]. However, many studies on body mass index (BMI) and mental illnesses have been cross-sectional [4–6], limiting assessment of temporal order. Some longitudinal studies have examined BMI and symptom changes in depression and anxiety [7, 8], but whether BMI influences the first-onset clinical diagnosis remains unclear. Additionally, underweight individuals were at increased risk of depression, yet their mental health needs were frequently overlooked in primary care settings [4]. There is little published evidence for associations between BMI and eating disorders or SMI, and few studies have examined underweight as a risk factor for mental disorders. The recent Lancet Commission recommended diagnosing clinical obesity using BMI, with excess adiposity confirmed via direct measures like waist-to-height ratio (WHtR) [9]. However, large-scale studies examining the association between WHtR and mental illnesses are still limited. Investigating whether obesity and underweight precede first-onset mental illness could inform prevention strategies.

Sex, age, and ethnicity may modify the association between BMI and mental illnesses. Genetic correlations between mental illnesses and body composition are sex-specific [10]. Obesity is more strongly associated with depression in women, possibly due to greater exposure to weight-related stigma [11]. An observational study of 4,932 adults indicated that older adults with obesity (BMI 30–35 kg/m^2^) had a significantly lower risk of depression than people with a healthy weight [7]. However, another longitudinal study that followed older adults’ BMI trajectories over five years found no significant association between the development of obesity and depression [12]. Research indicated that people from Asian ethnic groups have a greater tendency to store fat in the abdominal and visceral regions [13], which may contribute to an increased risk of mental illness [14]. Large-scale, long-term cohort studies are needed to identify related demographic patterns and associations and determine whether low BMI is also a risk factor.

Obesity increases the risk of cardiometabolic diseases (e.g., diabetes, cardiovascular disease [CVD]), which commonly co-occur with mental illness [15]. A study reported that the incidence of depression in individuals with diabetes was twice that of those without diabetes [16]. Another study showed that both CVD, including coronary artery disease and heart failure, and CVD risk factors, including high blood pressure, are prevalent among individuals with schizophrenia [17]. Although the association between metabolic pathways and mental illnesses have been discussed [18], the direction and longitudinal presentations remain incompletely understood. Obesity may increase mental illness risk through metabolic dysfunction yet few studies have tested this hypothesis.

To address these gaps, we used United Kingdom (UK) primary care records to conduct the largest longitudinal study to date on BMI and the first onset of diagnosed mental illness whilst controlling for potential mediating factors, including cardiometabolic disorders. Our study aimed to provide clinical insights into the associations between underweight and overweight and mental illness, informing early intervention and prevention strategies.

## Methods

### Study design, setting and participants

This population-based cohort study used data from the UK Clinical Practice Research Datalink (CPRD) Aurum database [19]. The CPRD is one of the world’s largest databases of primary care electronic health records, containing anonymised patient data from over 25% of the current UK population. CPRD has ethics approval from the Health Research Authority to support research using anonymised patient data [20]. Our study included 10,465,562 individuals aged 18 or older with a recorded BMI assessment between Jan 1, 2000 and Jan 1, 2022. Individuals provided written informed consent after receiving a complete description of the study. More details are described in Additional file 1 p1.

### Measurement of adiposity

BMI was calculated as weight (kg) divided by height (m^2^). Records without any measurements or BMI data outside of the plausible range (15–50 kg/m^2^) were excluded. Following WHO guidelines [21], BMI was classified as: underweight (< 18.5 kg/m^2^), normal (18.5–24.9 kg/m^2^), overweight (25.0–29.9 kg/m^2^), obesity class I (30.0–34.9 kg/m^2^), class II (35.0–39.9 kg/m^2^), and class III (≥ 40 kg/m^2^). Details on BMI data processing can be found in Additional file 1 p1.

WHtR was calculated by dividing the waist circumference by standing height. Referring to previous studies [22], central obesity was determined as WHtR ≥ 0.5. No thresholds for WHtR have been established for underweight. WHtR data processing were consistent with those for BMI (Additional file 1 p1).

From all available BMI and WHtR measurements, the closest recorded measurement to study entry was used and entered into the models.

### Ascertainment of mental illnesses

Mental illnesses were identified from linked records based on: 1) medical codes for depression, anxiety, eating disorders, bipolar disorder, schizophrenia, or other psychoses, as recorded in primary care (Additional file 1 p1), or 2) general practice referrals for treatment that implied a diagnosis, or 3) prescriptions of certain psychotropic medications where the prescription of a drug is very likely to indicate the occurrence of a specific mental illness. Many of these diagnoses were made by psychiatrists in secondary care using International Classification of Diseases (ICD) criteria and communicated to primary care physicians.

### Covariates

A directed acyclic graph guided covariate selection (Additional file 1 figure S1). We adjusted for baseline confounders: age, sex, self-reported ethnicity, general practice location, socioeconomic status, marital status, smoking status, drinking status, physical activity, weight-related comorbidities (CVD, hypertension, type 2 diabetes, chronic obstructive pulmonary disease) [23], obstructive sleep apnoea [24], renal failure [25], thyroid disease [26], hyperglycaemia [27], anaemia [28], multiple sclerosis [29], inflammatory bowel disease [30], eczema [31]. Covariate details are in Additional file 1 p1.

### Statistical analysis

#### Associations between BMI and mental illnesses

Baseline characteristics were presented as frequencies for categorical data, medians and interquartile range (IQR) for non-normally distributed continuous data, and mean and standard deviation (SD) for normally distributed continuous data. These data were presented for the whole cohort and stratified by BMI category. Missing data counts and percentages were reported.

After verifying the proportional hazards assumption using Schoenfeld residuals, Cox proportional hazard models were applied to estimate the hazard ratio (HR) and corresponding 95% confidence intervals (CIs) between the baseline BMI group and first-onset mental illnesses, with the healthy weight group (18.5–25 kg/m^2^) as the reference. Models were adjusted sequentially: model 1 (unadjusted), model 2 (age, sex), model 3 (demographics), model 4 (health behaviors), and model 5 (fully adjusted for weight-related comorbidities). For SMI outcomes, model 5 also adjusted for depression and anxiety. Follow-up ended at mental illness diagnosis, CPRD practice exit, death, or study period end, whichever came first. Restricted cubic spline models with five knots were used to assess non-linear associations in the fully adjusted model (model 5).

#### Mediation analysis

We excluded individuals with prevalent CVD, hypertension, or type 2 diabetes at baseline. Individuals with BMI ≥ 21 kg/m^2^ were included in this analysis. Mediators were defined as CVD, hypertension, or type 2 diabetes occurring after BMI measurement but before first onset of mental illnesses. Secondary analyses were performed to explore the potential mediating roles of inflammatory and cardiometabolic risk markers (C-reactive protein [CRP], systolic and diastolic blood pressure, triglycerides, low-density lipoprotein [LDL], total cholesterol, and hemoglobin A1c [HbA1c]), all measured after BMI assessment but before the first onset of mental illnesses. These markers were averaged across available measurements. We performed causal mediation analysis using the “mediation” package in R software [32], which estimates the proportional direct and indirect through 100 quasi-Bayesian simulations [33]. BMI was standardised to mean 0 and SD 1 for interpretation.

#### Sensitivity, exploratory, and heterogeneity analyses

First, to minimise reverse causality, we excluded the first 5 years of follow-up after the first BMI record in individuals with mental illness. Second, we also excluded individuals aged over 40 at cohort inception, as first episodes of mental illnesses mostly present in younger individuals [34]. Third, we restricted the analysis to individuals who had BMI records less than one year after registration, aiming to exclude patients who transferred practice and had a new code added to the medical record for the first time, rather than a new diagnosis. Fourth, to avoid confounding by pre-existing mental health conditions in patients with SMI, we excluded individuals with common mental illness at baseline and estimated the association between BMI and first-onset SMI. Finally, we used the date of diagnosis for SMI rather than symptoms as the incident date to test the effect of diagnosis time.

Some data on ethnicity, marital status, smoking, drinking, and physical activity were missing. We implemented 10 multiple imputations by chained equations, incorporating all variables from the fully adjusted model to mitigate bias.

We assessed heterogeneity in BMI-mental illnesses associations by sex (women/men), age (< 40, ≥ 40 years), and ethnicity. We also examined WHtR, categorising individuals as < 0.5 or ≥ 0.5.

Two-sided p-values < 0.05 were considered statistically significant. All analyses were performed using Stata version 16 and R version 3.4.2.

## Results

### Demographic characteristics

Of the total eligible population of 11,078,622 individuals, 10,465,562 were included in the study after excluding individuals diagnosed with dementia or cancer at baseline. For the analysis of each mental illness, those with a reported history of that illness at baseline were also excluded (Additional file 1 figure S2). The mean age was 45.8 (SD 17.9) years, and 58.6% were women (Table 1). During the study period, we identified 1,653,064 first diagnoses of depression, 919,177 of anxiety, 53,623 of anorexia nervosa, 6,050 of bulimia nervosa, 21,943 of other unspecified eating disorders, 25,555 of bipolar disorder, 26,341 of schizophrenia, and 13,645 of other psychoses, with a median follow-up period ranging from 5.5 to 6.9 years.

**Table 1:** Baseline characteristics of study population at the time of BMI measure by WHO BMI category in the CPRD

	**All patients** **(n = 10,465,562)**	**Underweight** **(< 18.5 kg/m**^**2**^**); n = 285**,**143**	**Healthy weight** **(18.5–25 kg/m**^**2**^**); n = 4,053,343**	**Overweight** **(25–30 kg/m**^**2**^**); n = 3,568,543**	**Obesity** **(30–35 kg/m**^**2**^**);** **n = 1,681,368**	**Obesity** **(35–40 kg/m**^**2**^**); n = 599,663**	**Obesity** **(**≥ **40.0 kg/m**^**2**^**); n = 277,502**
Age at baseline, years	45.8 (17.9)	38.4 (21.7)	42.5 (18.7)	48.7 (17.1)	48.6 (16.3)	46.6 (15.8)	44.6 (15.1)
Sex							
Women	6,128,668 (58.6%)	212,434 (74.5)	2,695,380 (66.5%)	1,770,591 (49.6%)	885,632 (52.7%)	371,165 (61.9%)	193,466 (69.7%)
Men	4,336,894 (41.4%)	72,709 (25.5%)	1,357,963 (33.5%)	1,797,952 (50.4%)	795,736 (47.3%)	228,498 (38.1%)	84,036 (30.3%)
Ethnicity							
White	6,835,920 (65.3%)	161,650 (56.7%)	2,605,625 (64.3%)	2,340,618 (65.6%)	1,124,178 (66.9%)	408,695 (68.2%)	195,154 (70.3%)
Asian	693,587 (6.6%)	27,934 (9.8%)	284,592 (7.0%)	249,260 (7.0%)	95,976 (5.7%)	26,646 (4.4%)	9,179 (3.3%)
Black	393,530 (3.8%)	7,419 (2.6%)	113,445 (2.8%)	142,205 (4.0%)	83,943 (5.0%)	31,914 (5.3%)	14,604 (5.3%)
Other	100,132 (1.0%)	2,811 (1.0%)	38,627 (1.0%)	34,814 (1.0%)	16,193 (1.0%)	5,510 (0.9%)	2,177 (0.8%)
Mixed	122,827 (1.2%)	3,941 (1.4%)	49,613 (1.2%)	39,677 (1.1%)	19,031 (1.1%)	7,048 (1.2%)	3,517 (1.3%)
Missing	2,319,566 (22.2%)	81,388 (28.5%)	961,441 (23.7%)	761,969 (21.4%)	342,047 (20.3%)	119,850 (20.0%)	52,871 (19.1%)
Marital status							
Single	431,867 (4.1%)	17,110 (6.0%)	197,651 (4.9%)	120,445 (3.4%)	58,427 (3.5%)	24,536 (4.1%)	13,698 (4.9%)
Married	670,259 (6.4%)	10,497 (3.7%)	228,382 (5.6%)	243,466 (6.8%)	123,167 (7.3%)	44,221 (7.4%)	20,526 (7.4%)
Widowed	119,281 (1.1%)	3,648 (1.3%)	39,922 (1.0%)	40,210 (1.1%)	21,978 (1.3%)	8,934 (1.5%)	4,589 (1.7%)
Divorced	65,072 (0.6%)	1,084 (0.4%)	19,931 (0.5%)	23,310 (0.7%)	13,192 (0.8%)	5,112 (0.9%)	2,443 (0.9%)
Separated	30,776 (0.3%)	543 (0.2%)	10,039 (0.2%)	10,646 (0.3%)	5,921 (0.4%)	2,424 (0.4%)	1,203 (0.4%)
Missing	9,148,307 (87.4%)	252,261 (88.5%)	3,557,418 (87.8%)	3,130,466 (87.7%)	1,458,683 (86.8%)	514,436 (85.8%)	235,043 (84.7%)
Townsend deprivation scores							
Quintile 1 (most affluent)	2,066,111 (19.7%)	47,605 (16.7%)	859,671 (21.2%)	735,358 (20.6%)	296,128 (17.6%)	91,333 (15.2%)	36,016 (13.0%)
Quintile 2	2,115,723 (20.2%)	52,056 (18.3%)	847,115 (20.9%)	738,151 (20.7%)	326,283 (19.4%)	106,857 (17.8%)	45,261 (16.3%)
Quintile 3	2,046,740 (19.6%)	53,982 (18.9%)	799,790 (19.7%)	699,891 (19.6%)	326,566 (19.4%)	114,666 (19.1%)	51,845 (18.7%)
Quintile 4	2,171,600 (20.7%)	62,840 (22.0%)	818,178 (20.2%)	726,099 (20.3%)	363,807 (21.6%)	135,489 (22.6%)	65,187 (23.5%)
Quintile 5	2,055,237 (19.6%)	68,394 (24.0%)	724,626 (17.9%)	665,598 (18.7%)	366,939 (21.8%)	150,755 (25.1%)	78,925 (28.4%)
Missing	10,151 (0.1%)	266 (0.1%)	3963 (0.1%)	3446 (0.1%)	1645 (0.1%)	563 (0.1%)	268 (0.1%)
Smoking status							
Non-smoker	3,961,930 (37.9%)	114,091 (40.0%)	1,626,047 (40.1%)	1,285,785 (36.0%)	604,118 (35.9%)	223,706 (37.3%)	108,183 (39.0%)
Ex-smoker	1,531,836 (14.6%)	21,706 (7.6%)	487,007 (12.0%)	577,857 (16.2%)	294,011 (17.5%)	103,631 (17.3%)	47,624 (17.2%)
Current-smoker	2,511,149 (24.0%)	80,525 (28.2%)	983,651 (24.3%)	835,253 (23.4%)	400,875 (23.8%)	144,461 (24.1%)	66,384 (23.9%)
Missing	2,460,647 (23.5%)	68,821 (24.1%)	956,638 (23.6%)	869,648 (24.4%)	382,364 (22.7%)	127,865 (21.3%)	55,311 (19.9%)
Alcohol use							
Non-drinker	750,803 (7.2%)	24,145 (8.5%)	271,553 (6.7%)	245,671 (6.9%)	130,485 (7.8%)	51,924 (8.7%)	27,025 (9.7%)
Ex-drinker	24,890 (0.2%)	753 (0.3%)	8,536 (0.2%)	7,932 (0.2%)	4,672 (0.3%)	1,946 (0.3%)	1,051 (0.4%)
Current-drinker	6,505,129 (62.2%)	145,847 (51.1%)	2,458,412 (60.7%)	2,271,700 (63.7%)	1,075,430 (64.0%)	380,207 (63.4%)	173,533 (62.5%)
Missing	3,184,740 (30.4%)	114,398 (40.1%)	1,314,842 (32.4%)	1,043,240 (29.2%)	470,781 (28.0%)	165,586 (27.6%)	75,893 (27.3%)
Physical activity							
Limited/light exercise	1,855,324 (17.7%)	45,209 (15.9%)	638,512 (15.8%)	624,466 (17.5%)	341,510 (20.3%)	136,157 (22.7%)	69,470 (25.0%)
Regular exercise	1,490,221 (14.2%)	30,111 (10.6%)	606,598 (15.0%)	522,417 (14.6%)	225,708 (13.4%)	73,950 (12.3%)	31,437 (11.3%)
Missing	7,120,017 (68.0%)	209,823 (73.6%)	2,808,233 (69.3%)	2,421,660 (67.9%)	1114,150 (66.3%)	389,556 (65.0%)	176,595 (63.6%)
COPD	230,403 (2.2%)	12,652 (4.4%)	84,176 (2.1%)	75,414 (2.1%)	37,943 (2.3%)	13,712 (2.3%)	6,506 (2.3%)
Obstructive sleep apnoea	24,815 (0.2%)	39 (0.0%)	1,498 (0.0%)	5,714 (0.2%)	7,311 (0.4%)	5,328 (0.9%)	4,925 (1.8%)
Renal failure	195,305 (1.9%)	5,014 (1.8%)	56,671 (1.4%)	71,675 (2.0%)	40,230 (2.4%)	14,762 (2.5%)	6,953 (2.5%)
Thyroid disease	420,608 (4.0%)	8,563 (3.0%)	130,504 (3.2%)	143,591 (4.0%)	83,279 (5.0%)	35,658 (5.9%)	19,013 (6.9%)
Hyperglycaemia	43,948 (0.4%)	311 (0.1%)	7,468 (0.2%)	15,617 (0.4%)	11,882 (0.7%)	5,375 (0.9%)	3,295 (1.2%)
Anaemia	350,172 (3.3%)	12,755 (4.5%)	136,183 (3.4%)	111,197 (3.1%)	56,466 (3.4%)	22,146 (3.7%)	11,425 (4.1%)
Multiple sclerosis	21,596 (0.2%)	838 (0.3%)	9,045 (0.2%)	6,835 (0.2%)	3,095 (0.2%)	1,217 (0.2%)	566 (0.2%)
Inflammatory bowel disease	500,153 (4.8%)	12,068 (4.2%)	200,809 (5.0%)	166,214 (4.7%)	78,763 (4.7%)	28,875 (4.8%)	13,424 (4.8%)
Eczema	1,283,435 (12.3%)	27,056 (9.5%)	477,476 (11.8%)	452,866 (12.7%)	213,756 (12.7%)	76,338 (12.7%)	35,943 (13.0%)

Data are n (%), mean (standard deviation), or median (interquartile range)

For variables with missing entries, category percentages are calculated out of participants with complete data (as shown by denominators), and percentages for missing data are calculated out of total participants

### The association between baseline BMI groups and first-onset mental illness

Compared with the healthy BMI category (18.5–25 kg/m^2^), both underweight (< 18.5 kg/m^2^) and obesity (≥ 30 kg/m^2^) were associated with a higher risk of incident mental illness in the fully adjusted model (Table 2). Underweight individuals had a higher risk of depression (HR 1.28; 95% CI 1.27–1.29), anxiety (1.26; 1.24–1.27), anorexia nervosa (3.88; 3.77–4.01), bulimia nervosa (1.52; 1.37–1.68), other unspecified eating disorders (4.50; 4.33–4.67), schizophrenia (1.46; 1.35–1.56), and other psychoses (1.22; 1.10–1.35), but no significant association with bipolar disorder (1.01; 0.94–1.10). Individuals with severe obesity (BMI ≥ 40 kg/m^2^) at baseline had a higher risk of depression (1.32; 1.31–1.33), anxiety (1.12; 1.10–1.13), bulimia nervosa (1.38; 1.20–1.58), other unspecified eating disorders (2.04; 1.91–2.17), bipolar disorder (1.44; 1.36–1.54), schizophrenia (2.02; 1.91–2.15), and other psychoses (1.40; 1.28–1.53). However, they had a lower risk of anorexia nervosa (0.45; 0.42–0.49).

**Table 2:** Adjusted associations between BMI category and mental illness

	**HR (95%CI) compared with healthy weight**					
**Mental illness**	**Underweight** **(< 18.5 kg/m**^**2**^**)**	**Healthy weight** **(18.5–25 kg/m**^**2**^**)**	**Overweight** **(25–30 kg/m**^**2**^**)**	**Obesity** **(30–35 kg/m**^**2**^**)**	**Obesity** **(35–40 kg/m**^**2**^**)**	**Obesity** **(**≥ **40 kg/m**^**2**^**)**
**Depression**						
Model 1 (unadjusted)	1.46 (1.44,1.47)	1.00 (REF)	0.88 (0.88,0.89)	1.00 (0.99,1.00)	1.20 (1.19,1.21)	1.46 (1.45,1.48)
Model 2 (model 1 plus age and sex)	1.32 (1.31,1.33)	1.00 (REF)	1.02 (1.01,1.02)	1.13 (1.12,1.14)	1.28 (1.27,1.29)	1.48 (1.47,1.50)
Model 3 (model 2 plus demographic factors)	1.31 (1.30,1.32)	1.00 (REF)	1.01 (1.01,1.02)	1.11 (1.10,1.11)	1.23 (1.22,1.23)	1.38 (1.37,1.40)
Model 4 (model 3 plus health behaviours)	1.30 (1.28,1.31)	1.00 (REF)	1.01 (1.01,1.01)	1.10 (1.09,1.10)	1.22 (1.21,1.23)	1.38 (1.37,1.39)
Model 5 (model 5 plus obesity-related morbidity)	1.28 (1.27,1.29)	1.00 (REF)	1.01 (1.01,1.01)	1.08 (1.08,1.09)	1.18 (1.17,1.19)	1.32 (1.31,1.33)
**Anxiety**						
Model 1 (unadjusted)	1.51 (1.49,1.52)	1.00 (REF)	0.79 (0.79,0.80)	0.85 (0.84,0.85)	0.99 (0.98,1.00)	1.17 (1.15,1.18)
Model 2 (model 1 plus age and sex)	1.26 (1.25,1.28)	1.00 (REF)	0.99 (0.98,0.99)	1.04 (1.03,1.04)	1.11 (1.10,1.12)	1.21 (1.20,1.23)
Model 3 (model 2 plus demographic factors)	1.27 (1.26,1.29)	1.00 (REF)	0.98 (0.98,0.99)	1.01 (1.01,1.02)	1.06 (1.05,1.07)	1.14 (1.13,1.15)
Model 4 (model 3 plus health behaviours)	1.26 (1.25,1.28)	1.00 (REF)	0.98 (0.97,0.98)	1.01 (1.00,1.02)	1.06 (1.05,1.07)	1.14 (1.12,1.15)
Model 5 (model 5 plus obesity-related morbidity)	1.26 (1.24,1.27)	1.00 (REF)	0.98 (0.98,0.98)	1.00 (1.00,1.01)	1.05 (1.04,1.06)	1.12 (1.10,1.13)
**Anorexia nervosa**						
Model 1 (unadjusted)	3.91 (3.79,4.03)	1.00 (REF)	0.73 (0.71,0.74)	0.68 (0.66,0.70)	0.62 (0.59,0.64)	0.54 (0.51,0.58)
Model 2 (model 1 plus age and sex)	4.43 (4.29,4.57)	1.00 (REF)	0.65 (0.63,0.66)	0.61 (0.60,0.63)	0.59 (0.56,0.61)	0.55 (0.51,0.59)
Model 3 (model 2 plus demographic factors)	4.06 (3.94,4.19)	1.00 (REF)	0.65 (0.63,0.66)	0.60 (0.58,0.62)	0.56 (0.53,0.58)	0.51 (0.48,0.55)
Model 4 (model 3 plus health behaviours)	3.96 (3.84,4.09)	1.00 (REF)	0.65 (0.64,0.66)	0.60 (0.59,0.62)	0.56 (0.53,0.58)	0.51 (0.48,0.55)
Model 5 (model 5 plus obesity-related morbidity)	3.88 (3.77,4.01)	1.00 (REF)	0.64 (0.62,0.65)	0.56 (0.55,0.58)	0.51 (0.49,0.53)	0.45 (0.42,0.49)
**Bulimia nervosa**						
Model 1 (unadjusted)	2.27 (2.06,2.51)	1.00 (REF)	0.44 (0.41,0.47)	0.45 (0.42,0.49)	0.68 (0.61,0.76)	1.05 (0.91,1.20)
Model 2 (model 1 plus age and sex)	1.44 (1.31,1.60)	1.00 (REF)	0.82 (0.77,0.88)	0.85 (0.78,0.93)	1.04 (0.93,1.16)	1.37 (1.20,1.57)
Model 3 (model 2 plus demographic factors)	1.50 (1.36,1.66)	1.00 (REF)	0.84 (0.79,0.90)	0.88 (0.80,0.96)	1.06 (0.95,1.19)	1.40 (1.22,1.60)
Model 4 (model 3 plus health behaviours)	1.52 (1.37,1.67)	1.00 (REF)	0.83 (0.78,0.89)	0.87 (0.80,0.95)	1.06 (0.95,1.19)	1.40 (1.22,1.61)
Model 5 (model 5 plus obesity-related morbidity)	1.52 (1.37,1.68)	1.00 (REF)	0.84 (0.78,0.89)	0.87 (0.80,0.95)	1.05 (0.94,1.18)	1.38 (1.20,1.58)
**Other unspecified eating disorders**						
Model 1 (unadjusted)	6.55 (6.31,6.80)	1.00 (REF)	0.39 (0.37,0.40)	0.49 (0.47,0.51)	0.89 (0.84,0.94)	1.73 (1.63,1.84)
Model 2 (model 1 plus age and sex)	4.36 (4.20,4.53)	1.00 (REF)	0.68 (0.65,0.70)	0.85 (0.81,0.89)	1.28 (1.21,1.35)	2.16 (2.04,2.30)
Model 3 (model 2 plus demographic factors)	4.50 (4.34,4.67)	1.00 (REF)	0.68 (0.66,0.71)	0.86 (0.82,0.90)	1.28 (1.20,1.35)	2.13 (2.00,2.26)
Model 4 (model 3 plus health behaviours)	4.53 (4.36,4.70)	1.00 (REF)	0.68 (0.65,0.71)	0.86 (0.82,0.90)	1.27 (1.20,1.35)	2.12 (2.00,2.26)
Model 5 (model 5 plus obesity-related morbidity)	4.50 (4.33,4.67)	1.00 (REF)	0.68 (0.65,0.71)	0.85 (0.81,0.89)	1.24 (1.17,1.32)	2.04 (1.91,2.17)
**Bipolar disorder**						
Model 1 (unadjusted)	1.21 (1.11,1.31)	1.00 (REF)	1.03 (1.00,1.06)	1.28 (1.23,1.32)	1.67 (1.60,1.75)	1.91 (1.80,2.04)
Model 2 (model 1 plus age and sex)	1.10 (1.02,1.20)	1.00 (REF)	1.15 (1.12,1.19)	1.41 (1.36,1.46)	1.77 (1.69,1.85)	1.94 (1.82,2.07)
Model 3 (model 2 plus demographic factors)	1.09 (1.01,1.19)	1.00 (REF)	1.14 (1.10,1.17)	1.36 (1.31,1.41)	1.66 (1.58,1.74)	1.76 (1.66,1.88)
Model 4 (model 3 plus health behaviours)	1.08 (0.99,1.17)	1.00 (REF)	1.14 (1.10,1.17)	1.35 (1.31,1.40)	1.65 (1.57,1.73)	1.75 (1.65,1.87)
Model 5 (model 4 plus obesity-related morbidity, depression, and anxiety)	1.01 (0.94,1.10)	1.00 (REF)	1.11 (1.07,1.14)	1.25 (1.21,1.30)	1.45 (1.38,1.52)	1.44 (1.36,1.54)
**Schizophrenia**						
Model 1 (unadjusted)	1.64 (1.53,1.76)	1.00 (REF)	1.11 (1.08,1.14)	1.58 (1.52,1.63)	2.06 (1.97,2.16)	2.40 (2.26,2.54)
Model 2 (model 1 plus age and sex)	1.70 (1.58,1.83)	1.00 (REF)	1.01 (0.98,1.04)	1.46 (1.41,1.52)	2.03 (1.94,2.12)	2.49 (2.35,2.64)
Model 3 (model 2 plus demographic factors)	1.57 (1.46,1.68)	1.00 (REF)	0.99 (0.96,1.02)	1.38 (1.33,1.42)	1.85 (1.77,1.93)	2.19 (2.07,2.32)
Model 4 (model 3 plus health behaviours)	1.50 (1.40,1.61)	1.00 (REF)	1.02 (0.99,1.05)	1.41 (1.36,1.46)	1.88 (1.80,1.97)	2.22 (2.09,2.36)
Model 5 (model 4 plus obesity-related morbidity, depression, and anxiety)	1.46 (1.35,1.56)	1.00 (REF)	1.01 (0.98,1.05)	1.38 (1.33,1.42)	1.79 (1.71,1.87)	2.02 (1.91,2.15)
**Other psychoses**						
Model 1 (unadjusted)	1.36 (1.23,1.51)	1.00 (REF)	1.05 (1.00,1.09)	1.29 (1.23,1.36)	1.59 (1.49,1.70)	1.73 (1.59,1.90)
Model 2 (model 1 plus age and sex)	1.35 (1.21,1.50)	1.00 (REF)	1.04 (1.00,1.09)	1.29 (1.23,1.36)	1.59 (1.49,1.70)	1.75 (1.60,1.91)
Model 3 (model 2 plus demographic factors)	1.30 (1.17,1.44)	1.00 (REF)	1.02 (0.97,1.06)	1.21 (1.15,1.27)	1.45 (1.36,1.55)	1.55 (1.42,1.70)
Model 4 (model 3 plus health behaviours)	1.27 (1.14,1.41)	1.00 (REF)	1.02 (0.98,1.07)	1.22 (1.16,1.28)	1.45 (1.36,1.55)	1.56 (1.42,1.70)
Model 5 (model 4 plus obesity-related morbidity, depression, and anxiety)	1.22 (1.10,1.35)	1.00 (REF)	1.01 (0.97,1.05)	1.17 (1.11,1.23)	1.36 (1.27,1.45)	1.40 (1.28,1.53)

Model 5 adjusted for age, sex, general practitioner, ethnicity, socioeconomic status, marital status, smoking status, drinking status, physical activity, cardiovascular disease, type 2 diabetes, hypertension, chronic obstructive pulmonary disease, obstructive sleep apnoea, renal failure, thyroid disease, hyperglycaemia, anaemia, multiple sclerosis, inflammatory bowel disease, and eczema

### The association between baseline continuous BMI and first-onset mental illness

**Fig. 1:**
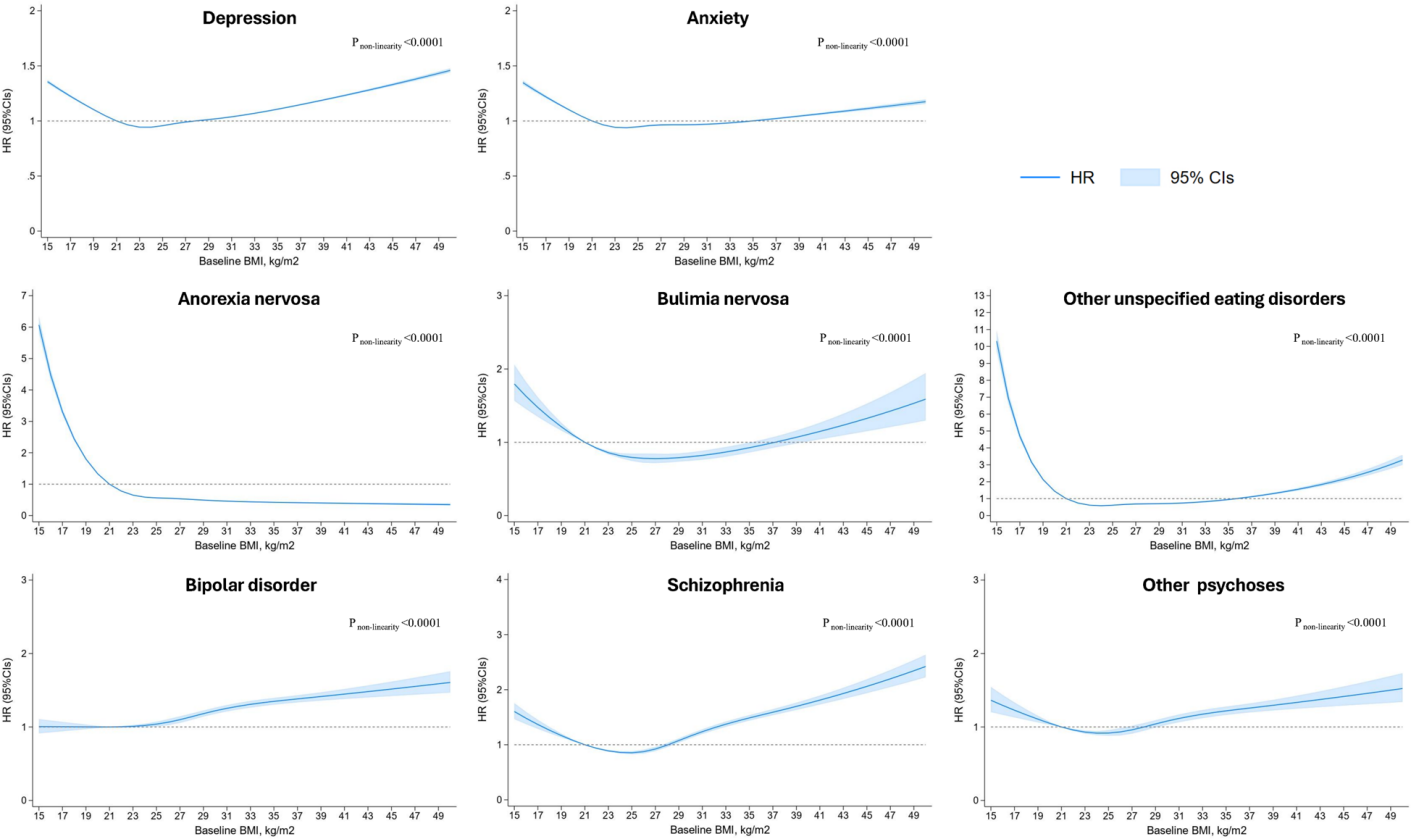
Associations between baseline BMI and mental illness in the total population. Estimates adjusted for age, sex, general practitioner, ethnicity, socioeconomic status, marital status, smoking status, drinking status, physical activity, cardiovascular disease, type 2 diabetes, hypertension, chronic obstructive pulmonary disease, obstructive sleep apnoea, renal failure, thyroid disease, hyperglycaemia, anaemia, multiple sclerosis, inflammatory bowel disease, and eczema. HR: hazard ratio, CIs: confidence intervals

There was a positive association between BMI above the healthy range and risks of diagnosed common and serious mental illnesses, except anorexia nervosa, where a decrease in risk was observed (Fig. 1). For bulimia nervosa and other unspecified eating disorders, an increase in risk was observed at low BMI, but minimal elevation in risk at higher BMI. The *p* values for non-linearity were less than 0.0001 for all outcomes.

### The association between baseline WHtR groups and first-onset mental illness

In exploratory analyses, compared with those with healthy or increased WHtR (< 0.5), individuals with high WHtR had a higher risk of depression (1.08; 1.06–1.09), anxiety (1.01; 1.00–1.03), bipolar disorder (1.21; 1.09–1.35), schizophrenia (1.42; 1.27–1.60), and other psychoses (1.34; 1.15–1.55); and a lower risk of anorexia nervosa (0.82; 0.76–0.87). We found no evidence of association of WHtR with bulimia nervosa and other unspecified eating disorders (Table 3).

**Table 3:** Adjusted associations between WHtR and mental illness

	**WHtR (**≥ **0.5)**	
**Mental illness**	**HR**	**95%CI**
**Depression**		
Model 1 (unadjusted)	1.01	1.00,1.02
Model 2 (model 1 plus age and sex)	1.13	1.12,1.15
Model 3 (model 2 plus demographic factors)	1.12	1.11,1.14
Model 4 (model 3 plus health behaviours)	1.11	1.10,1.13
Model 5 (model 5 plus obesity-related morbidity)	1.08	1.06,1.09
**Anxiety**		
Model 1 (unadjusted)	0.83	0.81,0.84
Model 2 (model 1 plus age and sex)	1.01	1.00,1.03
Model 3 (model 2 plus demographic factors)	1.02	1.00,1.04
Model 4 (model 3 plus health behaviours)	1.02	1.00,1.03
Model 5 (model 5 plus obesity-related morbidity)	1.01	1.00,1.03
**Anorexia nervosa**		
Model 1 (unadjusted)	1.17	1.09,1.25
Model 2 (model 1 plus age and sex)	0.94	0.88,1.00
Model 3 (model 2 plus demographic factors)	0.88	0.82,0.94
Model 4 (model 3 plus health behaviours)	0.87	0.81,0.93
Model 5 (model 5 plus obesity-related morbidity)	0.82	0.76,0.87
**Bulimia nervosa**		
Model 1 (unadjusted)	0.46	0.35,0.61
Model 2 (model 1 plus age and sex)	0.83	0.63,1.09
Model 3 (model 2 plus demographic factors)	0.86	0.65,1.15
Model 4 (model 3 plus health behaviours)	0.87	0.66,1.16
Model 5 (model 5 plus obesity-related morbidity)	0.86	0.65,1.15
**Other unspecified eating disorders**		
Model 1 (unadjusted)	0.56	0.49,0.64
Model 2 (model 1 plus age and sex)	0.91	0.80,1.04
Model 3 (model 2 plus demographic factors)	0.93	0.82,1.06
Model 4 (model 3 plus health behaviours)	0.94	0.82,1.07
Model 5 (model 5 plus obesity-related morbidity)	0.91	0.80,1.04
**Bipolar disorder**		
Model 1 (unadjusted)	1.18	1.06,1.31
Model 2 (model 1 plus age and sex)	1.38	1.24,1.54
Model 3 (model 2 plus demographic factors)	1.33	1.19,1.49
Model 4 (model 3 plus health behaviours)	1.32	1.19,1.48
Model 5 (model 4 plus obesity-related morbidity, depression, and anxiety)	1.21	1.09,1.35
**Schizophrenia**		
Model 1 (unadjusted)	1.60	1.43,1.79
Model 2 (model 1 plus age and sex)	1.67	1.48,1.87
Model 3 (model 2 plus demographic factors)	1.51	1.35,1.70
Model 4 (model 3 plus health behaviours)	1.51	1.35,1.70
Model 5 (model 4 plus obesity-related morbidity, depression, and anxiety)	1.42	1.27,1.60
**Other psychoses**		
Model 1 (unadjusted)	1.43	1.24,1.65
Model 2 (model 1 plus age and sex)	1.57	1.35,1.82
Model 3 (model 2 plus demographic factors)	1.43	1.24,1.66
Model 4 (model 3 plus health behaviours)	1.42	1.22,1.65
Model 5 (model 4 plus obesity-related morbidity, depression, and anxiety)	1.34	1.15,1.55

*WHtR *waist-to-height ratio

Model 5 adjusted for age, sex, general practitioner, ethnicity, socioeconomic status, marital status, smoking status, drinking status, physical activity, cardiovascular disease, type 2 diabetes, hypertension, chronic obstructive pulmonary disease, obstructive sleep apnoea, renal failure, thyroid disease, hyperglycaemia, anaemia, multiple sclerosis, inflammatory bowel disease, and eczema.

### Subgroup analyses

In heterogeneity analyses, both high and low BMI was more strongly associated with mental illness in women than in men except for depression, anxiety, bulimia nervosa, and other unspecified eating disorders (Additional file 1 figure S3). Most associations between BMI and mental illness were attenuated at older ages (Additional file 1 figure S4). We found no evidence of effect modification by age for bipolar disorder or other psychoses. In the results stratified by ethnicity, most associations between BMI and mental illness were higher in the Asian ethnic groups at higher BMI (Additional file 1 figure S5).

### Mediation analyses between excess weight and mental illness

Cardiometabolic disorders were not found to mediate the associations between BMI and common or severe mental illnesses (Additional file 1 table S1). Similarly, no mediating role was observed for the inflammatory marker CRP (Additional file 1 table S2). Among the cardiometabolic risk markers, low-density lipoprotein (LDL) and triglycerides were found to significantly mediate the associations between BMI and incident depression, anxiety, bipolar disorder, schizophrenia, and other psychoses (indirect effects ranging from 0.2% to 15%, p < 0.001). Cholesterol only mediated the association with other unspecified eating disorders (indirect effects 0.3%, 95% CI: 0.2%−0.5%, p < 0.001). Notably, systolic and diastolic blood pressure mediated the association with anorexia nervosa (indirect effects ranging from 18.2% to 18.8%, p < 0.001), while HbA1c mediated the associations with depression, other unspecified eating disorders, schizophrenia, and other psychoses (indirect effects ranging from 2.3% to 10.3%, p < 0.001).

### Sensitivity analyses

Our main findings were robust across sensitivity analyses, with similar estimated effect sizes in all scenarios. Details on findings have been described in Additional file 1 p2.

## Discussion

In this longitudinal study of over 10 million UK adults, both obesity and underweight BMI were associated with a higher first-onset of common and serious mental illnesses. BMI above the healthy range was linked to risks of common and serious mental illnesses, except anorexia nervosa, where a decrease in risk was observed. Underweight individuals showed increased risks of depression, anxiety, eating disorders, schizophrenia and other psychoses, with no association with bipolar disorder. The associations between obesity and depression, anxiety, and schizophrenia were more pronounced in women, younger individuals, and Asian ethnic groups. Metabolic disorders such as CVD, hypertension, and diabetes did not mediate these associations, though certain cardiometabolic markers (LDL, triglycerides, cholesterol, blood pressure, and HbA1c) were identified as mediators.

Some studies have investigated the association between BMI and depression risk, but there is less evidence for anxiety, eating disorders and serious mental illness. A cross-sectional study and a 7-year prospective cohort study indicated that both underweight and severe obesity were associated with an increased risk of depression [4, 35]. Our findings support this observation. BMI is an important objective indicator to evaluate nutritional status. The association between low BMI and mental illness may be attributed to multiple factors, such as frailty associated with underlying physical health conditions and nutritional deficiencies that support brain function [36]. A Netherlands cohort study found that obesity associated with a threefold increased anxiety risk at a three-year follow-up [6]. However, anxiety was assessed using a self-reported scale. For schizophrenia, the HR associated with severe obesity is highly clinically meaningful. A meta-analysis found that cigarette smoking is associated with an approximately 1.84-fold increased risk of developing schizophrenia, a highly comparable effect size [37]. This positions severe obesity as a risk factor of similar clinical importance to smoking in the etiology of schizophrenia. Directly comparable modifiable behavioral risk factors for anorexia nervosa of a similar magnitude are scarce in the literature. The high HR for underweight underscores that low BMI itself is an exceptionally strong and readily measurable indicator of risk, potentially one of the most potent identifiable markers for anorexia nervosa onset. This finding highlights the critical importance of monitoring BMI as a key component in early detection and prevention strategies. Our study showed that both underweight and obesity were significantly associated with an increased risk of diagnosed common and serious mental illness. This addresses the gap in using large-scale, long-term cohort studies on this topic and provides evidence associated with diagnosed mental disorders.

The stronger BMI-mental illness associations in women and younger participants (age < 40) align with previous research [10, 11]. A Mendelian randomization study showed genetically predicted BMI positively associated with depression, with stronger effects in women and younger individuals [11]. Similarly, Wootton et al. found higher BMI causally associated with lower subjective well-being [38]. Studies have shown that individuals with obesity are often subjected to weight-related stigma and discrimination, and women tend to be more vulnerable to stigmatization than men [39]. In our study, body image concerns likely contribute to stronger obesity-mental illness associations in younger women. In addition, studies showed that mental illness often occurs during adolescence and continues into adulthood, younger onset is associated with more episodes and admission to hospital [40, 41]. We also found that the risks associated with higher BMI were greater in Asian ethnic groups. The observed stronger association may reflect differences in help-seeking behaviors or cultural reporting of mental health symptoms across ethnic groups. In Asian societies, stronger social pressure regarding body weight may contribute to greater psychological distress in individuals with either low or high BMI [42]. The underlying mechanisms warrant further investigation and call for tailored public health interventions.

Our mediation analyses showed that cardiometabolic diseases did not mediate BMI-mental illness associations. So far, the mediating role of cardiometabolic diseases in obesity and mental illnesses has not yet been explored, but their associations have been a focus of study. Nong et al. collected data on a total of 33,001 individuals in 5 cycles of the National Health and Nutrition Examination Survey and found that obesity explained 28.6% of the risk of depression in people with diabetes [43]. Benjamin et al. conducted a cohort study of 14,975 individuals, using data from participants aged 1 to 24 [44]. They did not find consistent evidence for associations of fasting insulin level trajectories with depression. Another two-sample Mendelian randomization study showed that BMI had an impact on the direct effect of liability to schizophrenia on CVD [17]. While mental illness often manifested early, cardiometabolic risks may progressively accumulate afterwards. In the UK, people with SMI are offered an annual health check focused on cardiovascular prevention. Only about 35% of people on the UK primary care severe mental illness register received annual health checks at the end of 2021 [45]. This indicated insufficient health monitoring in early intervention services, particularly for managing cardiometabolic risks.

Our study showed that LDL, triglycerides, cholesterol, blood pressure, and HbA1c demonstrated partial mediation (< 15%), While this degree of mediation may have limited clinical relevance, it is nonetheless plausible in light of previous studies. Although no prior mediation analyses were available on this topic, two previous Mendelian randomization studies suggested a consistent association between triglycerides and the risk of depression and schizophrenia [46, 47]. A Swedish cohort study including over 200,000 participants found that elevated levels of glucose and triglycerides, along with reduced high-density lipoprotein, were associated with an increased risk of developing depression, anxiety, and stress-related disorders [48]. The English Longitudinal Study of Ageing reported that the likelihood of depressive symptoms increased with higher HbA1c levels [49]. Previous evidence has also indicated that obesity is closely linked to dyslipidemia and dysglycemia [50, 51]. Notably, triglyceride levels have been shown to be inversely associated with cortical thickness in the rostral anterior cingulate cortex (rACC) [52]. The anterior cingulate cortex occupied a key position in the brain and was involved in emotional evaluation, emotion-related learning, and autonomic regulation [53]. Elevated triglycerides may thus exert adverse effects on rACC thickness. The mechanisms involving other biomarkers demand further exploration.

## Strength and limitations

This study has several strengths. Using UK primary care records, we conducted the largest longitudinal study to date on BMI and first-onset mental illness. Mental illness was comprehensively defined, incorporating diagnostic codes, medication records, and mental health referrals. Furthermore, the large sample also allowed us to assess the effect modification of the BMI risk by sex, age, and ethnicity. Several limitations should also be considered. Firstly, BMI is an incomplete surrogate for adiposity distribution or degree of visceral adiposity. However, consistent results were observed with WHtR in sensitivity analyses. Secondly, due to inherent limitations in the completeness of data recorded in primary care, some data were missing on ethnicity, marital status, smoking, drinking, and physical activity. However, we conducted an imputation during the sensitivity analysis, and the results remained stable after the imputation, suggesting that major bias is unlikely. Additionally, as with any observational analyses, residual confounding due to unmeasured covariates such as population density and occupation might have occurred. Lastly, although the main results were unchanged after excluding participants with a history of diagnosed mental illness and those diagnosed wihtin the first 5 years of follow-up, the possibility of reverse causality cannot be fully rule out, especially in relation to eating disorders and medication-induced weight changes.

## Conclusions

In conclusion, individuals with unhealthy weight status, including both obesity and underweight, are at increased risk of first-onset common and serious mental illnesses. There was a positive association between excess weight and the onset of common and serious mental illnesses, except anorexia nervosa, where a decrease in risk was observed. In addition, individuals with underweight showed an increased risk of first-onset mental illnesses, with the exception of bipolar disorder. The associations of obesity with depression, anxiety, and schizophrenia were more pronounced in women, younger individuals, and Asian ethnic groups. Cardiometabolic disorders were not found to mediate the association between obesity and mental illness.

## Supplementary Information

Additional file 1. Figures S1-S5. FigS1 - Directed Acyclic Graph. FigS2 - Flow diagram of study participants. FigS3 - Associations between baseline BMI and mental illness by sex group. FigS4 - Associations between baseline BMI and mental by age. FigS5 - Associations between baseline BMI and mental illness by ethnicity. Table S1-S9. Table S1 - Causal mediation analysis to assess the mediation effect of cardiometabolic disease for observed associations between BMI and incident mental illness risk. Table S2 - Causal mediation analysis to assess the mediation effect of inflammatory and cardiometabolic risk markers for observed associations between BMI and incident mental illness risk. Table S3 - Adjusted associations between BMI category and mental illness excluding the first 5 years of follow-up after the BMI record in individuals with mental illness. Table S4 - Adjusted associations between BMI category and mental illness after excluding individuals over 40 years old at baseline. Table S5 - Adjusted associations between BMI category and mental illness excluding individuals with cardiovascular disease, hypertension, and type 2 diabetes at baseline. Table S6 - Adjusted associations between BMI category and mental illness in individuals who had a BMI record less than 1 year after registration. Table S7 - Adjusted associations between BMI category and SMI using the diagnosis date of SMI as the incident date. Table S8 - Adjusted associations between BMI category and SMI excluding individuals with common mental illness at baseline. Table S9 - Adjusted associations between BMI category and mental illness after imputation.

## Data Availability

Electronic health records are, by definition, considered to be sensitive data in the UK by the Data Protection Act and cannot be shared via public deposition because of information governance restrictions in place to protect patient confidentiality. Access to data is available only once approval has been obtained through the individual constituent entities controlling access to the data. The primary care data can be requested via the application to 10.48329/zrvz-6a47. Codelists are available from the corresponding author.

## Abbreviations

SMI: Serious Mental Illnesses
DALYs: Disability-adjusted Life Years
BMI: Body Mass Index
WHtR: Waist-to-height Ratio
CVD: Cardiovascular Disease
UK: United Kingdom
CPRD: Clinical Practice Research Datalink
ICD: International Classification of Diseases
IQR: Interquartile Range
HR: Hazard Ratio
Cis: Confidence Intervals
CRP: C-reactive Protein
LDL: Low-density Lipoprotein
HbA1c: Hemoglobin A1c
rACC: Rostral Anterior Cingulate Cortex
