# Supplementary material for: Adiposity and the first-onset of diagnosed mental illnesses: a population-based cohort study of 10 million UK adults
Source: BMC Med. 2025 Nov 29;24:5. doi: 10.1186/s12916-025-04514-z (PMC12763996; doi:10.1186/s12916-025-04514-z)
Supplement: Supplementary file 1 — Additional file 1. Figures S1-S5. FigS1 - Directed Acyclic Graph. FigS2 - Flow diagram of study participants. FigS3 - Associations between baseline BMI and mental illness by sex group. FigS4 - Associations between baseline BMI and mental by age. FigS5 - Associations between baseline BMI and mental illness by ethnicity. Table S1-S9. Table S1 - Causal mediation analysis to assess the mediation effect of cardiometabolic disease for observed associations between BMI and incident mental illness risk. Table S2 - Causal mediation analysis to assess the mediation effect of inflammatory and cardiometabolic risk markers for observed associations between BMI and incident mental illness risk. Table S3 - Adjusted associations between BMI category and mental illness excluding the first 5 years of follow-up after the BMI record in individuals with mental illness. Table S4 - Adjusted associations between BMI category and mental illness after excluding individuals over 40 years old at baseline. Table S5 - Adjusted associations between BMI category and mental illness excluding individuals with cardiovascular disease, hypertension, and type 2 diabetes at baseline. Table S6 - Adjusted associations between BMI category and mental illness in individuals who had a BMI record less than 1 year after registration. Table S7 - Adjusted associations between BMI category and SMI using the diagnosis date of SMI as the incident date. Table S8 - Adjusted associations between BMI category and SMI excluding individuals with common mental illness at baseline. Table S9 - Adjusted associations between BMI category and mental illness after imputation. [file 12916_2025_4514_MOESM1_ESM.docx]

**Supplement materials**

**Method**

**Study design, setting and participants**

The CPRD population generally represents the UK population in terms of age and sex. Data collected from CPRD comprise demographic information; clinical diagnoses; records of clinical measurements, including body mass index (BMI), body weight, and height records taken during primary care consultations; prescriptions; clinical diagnosis of medical conditions; and referrals to specialist services.

**Measurement of adiposity**

We evaluated all records of body weight, height, and BMI from the general practice medical records, and computed additional BMI values from recorded weight and height, permitting a greater number of participants to be represented in BMI measurement. We preferentially use coded BMI values. If no coded value, where height and weight are recorded on the same date, we used these to calculate BMI. Remaining weight records with an older height record available are converted to BMI using the most recent height data, and remaining weight records with future height records available are converted to BMI using the next available height record.

Where waist and height are recorded on the same date, these were used to calculate WHtR. The remaining waist records with an older height record available were converted to WHtR using the most recent height data, and remaining waist records with future height records available were converted to WHtR using the next available height record.

From all available BMI and WHtR measurements, the closest recorded measurement to study entry was used and entered into the models.

**Ascertainment of mental illnesses**

We identified patients in the CPRD diagnosed with mental illness between 1 January 2000 and January 2022. The incident date of depression was retained as the earliest date of diagnosis, general practice referral, or prescriptions for antidepressants, whichever came first. The incident dates of anxiety and eating disorders were retained as the earliest date of diagnosis or general practice referral. The incident dates of SMI (bipolar disorder, schizophrenia, or other psychoses) were retained as the earliest date of SMI symptoms, diagnosis, or general practice referral, whichever came first. The date of mental illness was retained as the first date of diagnosis, general practice referrals, or prescriptions for psychotropic medications, whichever came first.

**Covariates**

We adjusted for the following confounders at baseline: age; sex; self-reported ethnicity (classified as White, Asian, Black, Mixed, and other ethnic groups); general practice, socioeconomic status (classified into quintiles using the Townsend score, an area-based deprivation score); marital status (classified as single, married, widowed, divorced, separated); smoking status (divided into non-smoker, ex-smoker, or current-smoker); drinking status (divided into non-drinker, ex-drinker, or current-drinker); physical activity (divided into undertaking limited/light exercise, or regular exercise) ; weight-related comorbidities such as CVD, hypertension, type 2 diabetes, chronic obstructive pulmonary disease, obstructive sleep apnoea, renal failure, thyroid disease, hyperglycaemia, anaemia, multiple sclerosis, inflammatory bowel disease, eczema. Classifications of presence or absence of diseases were recorded through clinical codes, or if the participant had been referred to specialist services for management of any of these conditions. Missing data on any of these confounders were coded as “unknown” and entered as a separate category.

**Results**

**Sensitivity analyses**

Excluding the first 5 years of follow-up time (N=9,446,753) slightly attenuated the associations between BMI and most outcomes (Table S3); After excluding individuals over 40 years old (N=4,465,258), a minimal decrease in risk was observed at low BMI, while a small elevation in risk at higher BMI (Table S4). Excluding individuals with cardiovascular disease, hypertension, and type 2 diabetes in baseline (N=8,027,168), as well as analyses restricted to individuals who had a BMI record less than 1 year after registration (N=3,489,214), yielded results similar to our main findings (Tables S5-S6). Using the diagnosis date of SMI as the incident date slightly attenuated associations between BMI and SMI (Table S7); After excluding individuals with common mental illness (N=8,906,808), a minimal increase in risk was observed at low BMI (Table S8). When we accounted for missing data among the covariates through multiple imputation, the associations between BMI and mental illness remained the same as in the primary analysis (Table S9).


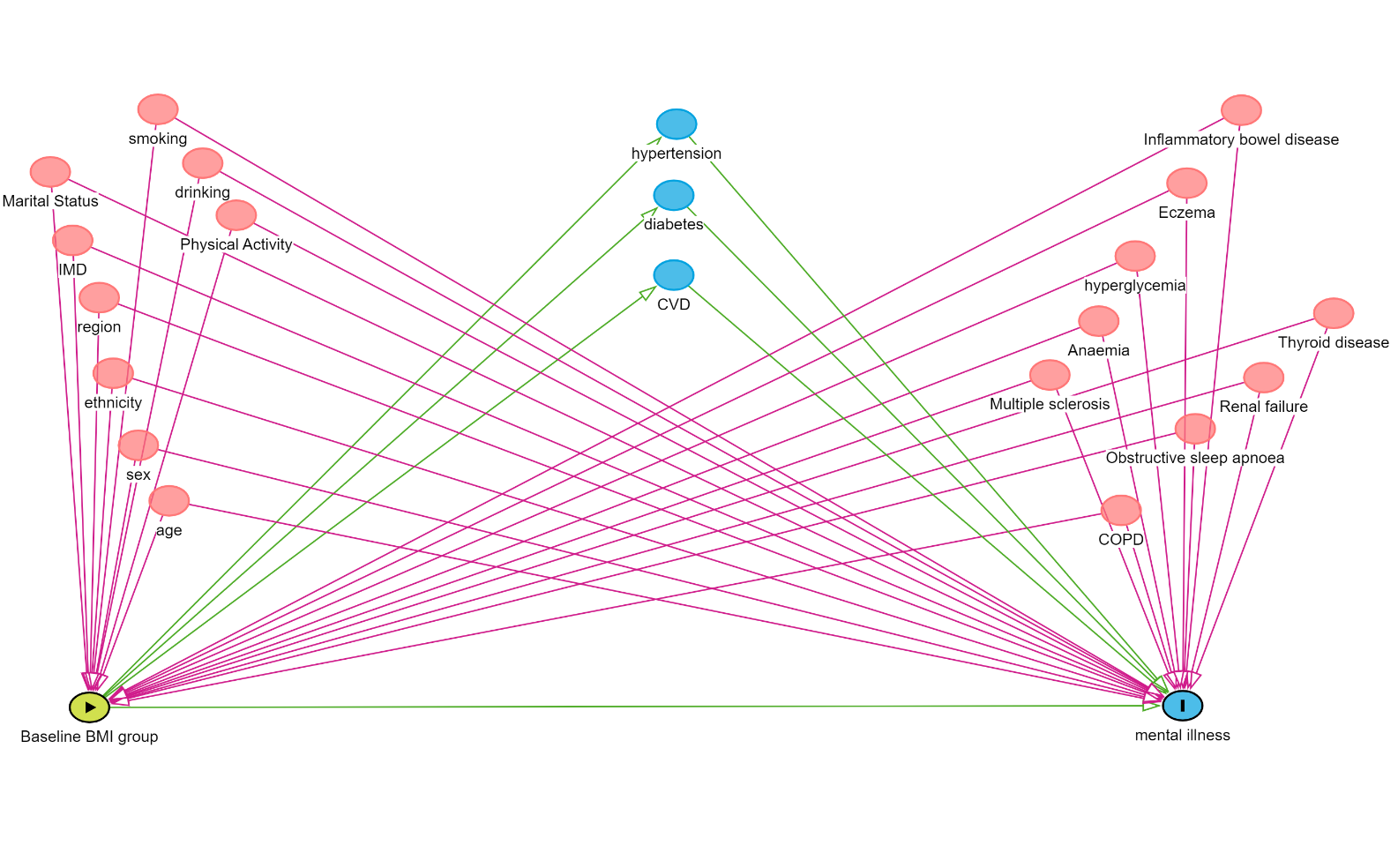


**Figure S1.** Directed Acyclic Graph

Green node = exposure; Blue node = outcome; Red node = adjusted set of variables; Green arrow = causal pathway; Red arrow = biasing pathway (confounding).

Mediators: CVD, hypertension, and diabetes.


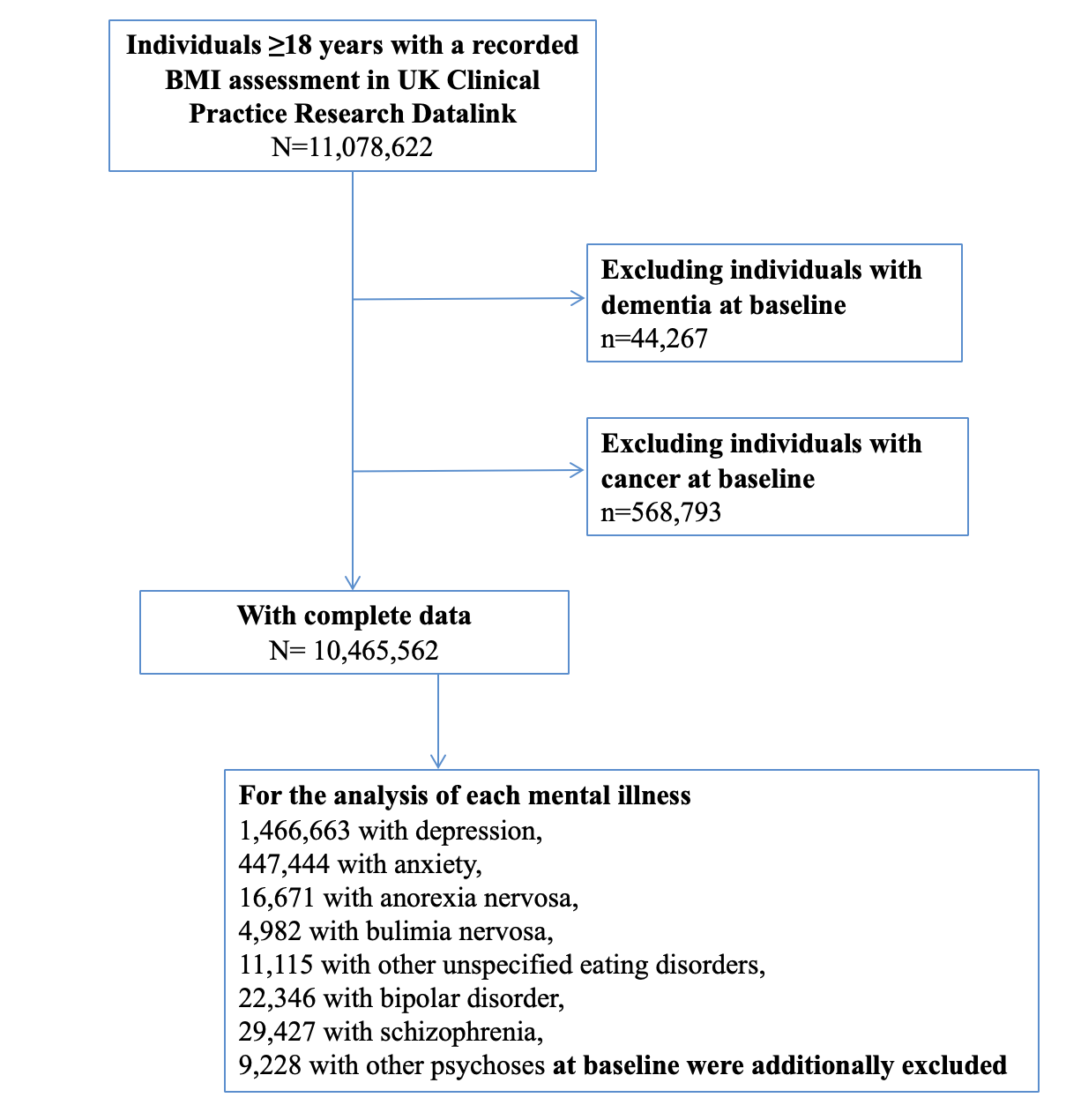


**Figure S2.** Flow diagram of study participants


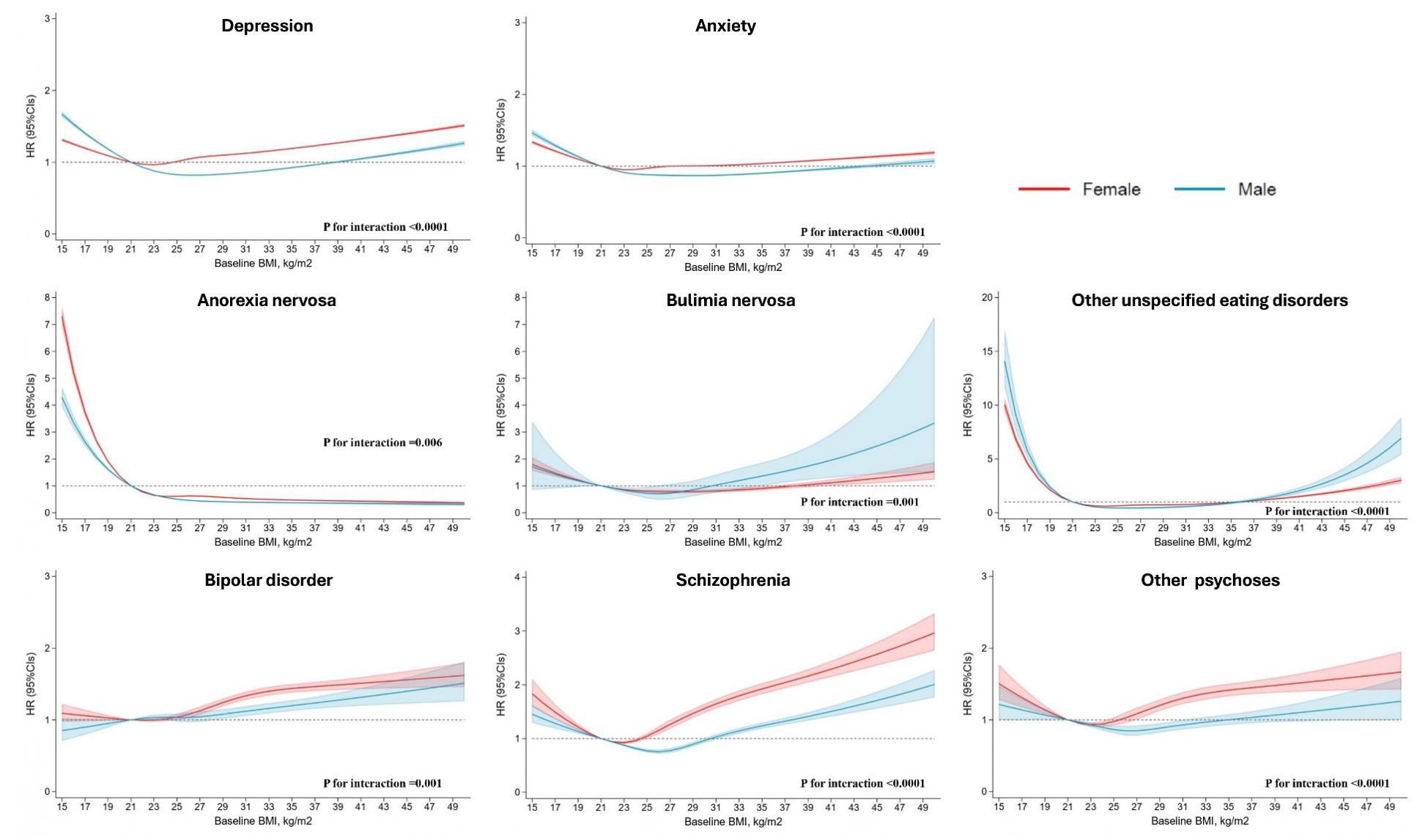


**Figure S3.** Associations between baseline BMI and mental illness by sex group

The solid line is the hazard ratio estimate, with shaded areas showing 95% CIs.

Estimates adjusted for age, general practice, ethnicity, socioeconomic status, region; marital status, smoking status, drinking status, physical activity, cardiovascular disease, type 2 diabetes, hypertension, chronic obstructive pulmonary disease, obstructive sleep apnoea, renal failure, thyroid disease, hyperglycaemia, anaemia, multiple sclerosis, inflammatory bowel disease, and eczema. HR=hazard ratio.


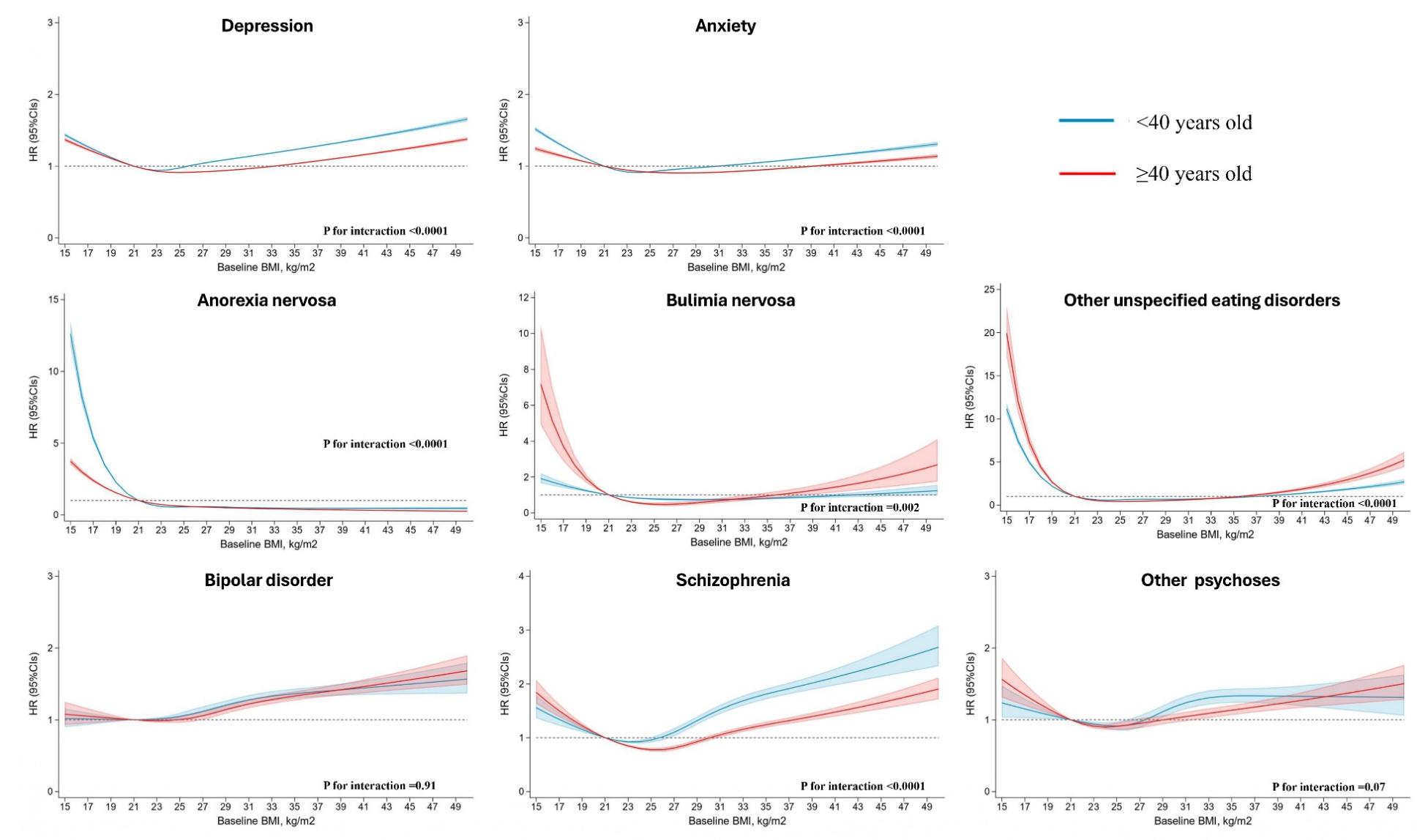


**Figure S4.** Associations between baseline BMI and mental by age

The solid line is the hazard ratio estimate, with shaded areas showing 95% CIs.

Estimates adjusted for sex, general practice, ethnicity, socioeconomic status, region; marital status, smoking status, drinking status, physical activity, cardiovascular disease, type 2 diabetes, hypertension, chronic obstructive pulmonary disease, obstructive sleep apnoea, renal failure, thyroid disease, hyperglycaemia, anaemia, multiple sclerosis, inflammatory bowel disease, and eczema. HR=hazard ratio.


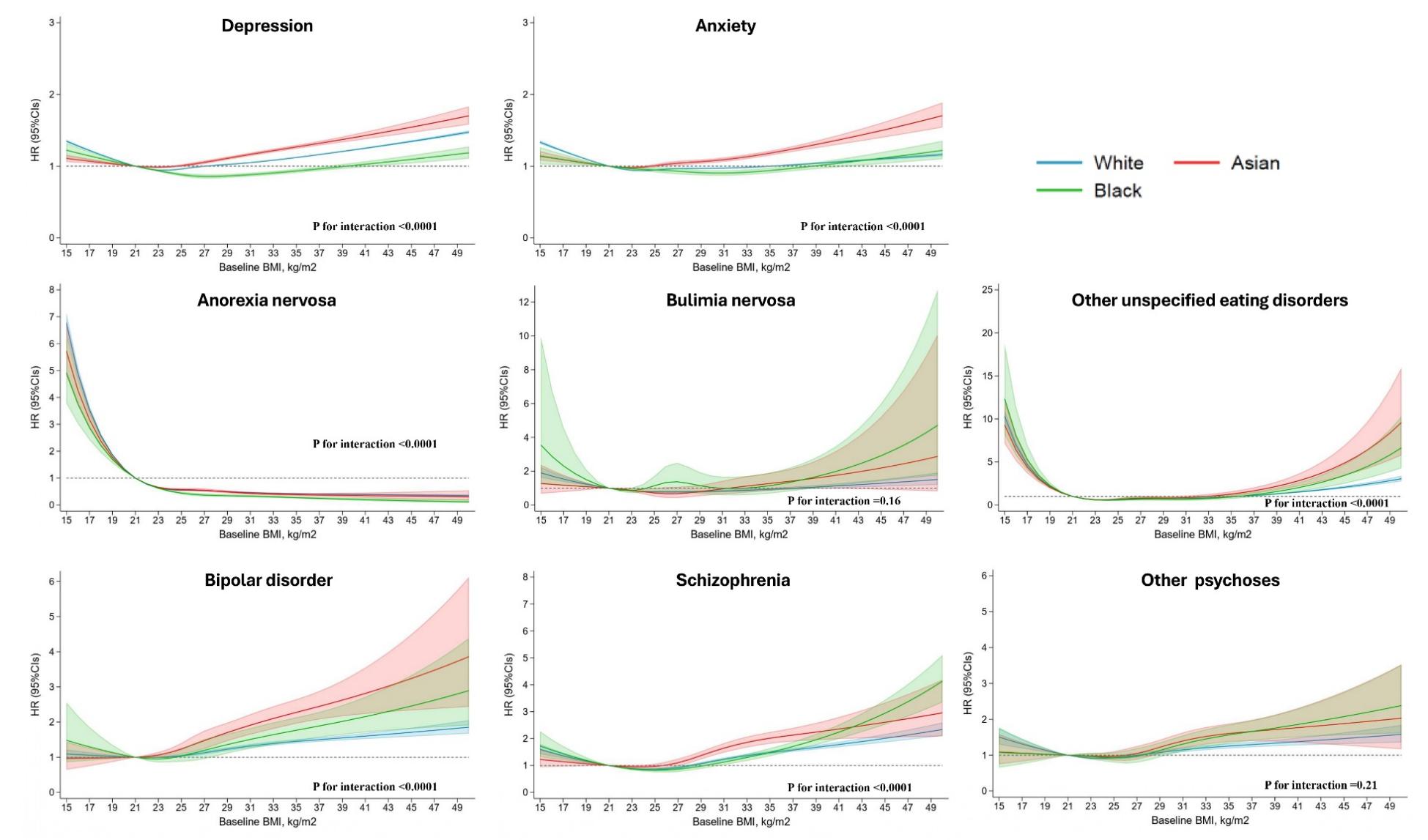


**Figure S5.** Associations between baseline BMI and mental illness by ethnicity

The solid line is the hazard ratio estimate, with shaded areas showing 95% CIs.

Estimates adjusted for age, sex, general practice, socioeconomic status, region; marital status, smoking status, drinking status, physical activity, cardiovascular disease, type 2 diabetes, hypertension, chronic obstructive pulmonary disease, obstructive sleep apnoea, renal failure, thyroid disease, hyperglycaemia, anaemia, multiple sclerosis, inflammatory bowel disease, and eczema. HR=hazard ratio.

**Table S1.** Causal mediation analysis to assess the mediation effect of cardiometabolic disease for observed associations between BMI and incident mental illness risk.

|  | **Total effect HR** | **Direct effect HR** | **Mediated HR** | **Proportion of Mediated effect (%)** |  |
| --- | --- | --- | --- | --- | --- |
| **Cardiovascular disease** |  |  |  |  |  |
| Depression | 1.08 (1.08,1.09) | 1.08 (1.08,1.09) | 0.59 (0.59,0.60) | - |  |
| Anxiety | 1.03 (1.02,1.03) | 1.03 (1.03,1.04) | 0.46 (0.46,0.47) | - |  |
| Bulimia nervosa | 1.05 (1.02,1.08) | 1.05 (1.02,1.09) | 0.23 (0.15,0.35) | - |  |
| Other unspecified eating disorders | 1.23 (1.21,1.25) | 1.23 (1.22,1.25) | 0.58 (0.51,0.66) | - |  |
| Bipolar disorders | 1.12 (1.10,1.13) | 1.12 (1.11,1.14) | 0.29 (0.27,0.32) | - |  |
| Schizophrenia | 1.26 (1.24,1.27) | 1.26 (1.25,1.28) | 0.22 (0.20,0.24) | - |  |
| Other psychoses | 1.12 (1.10,1.15) | 1.13 (1.11,1.15) | 0.31 (0.28,0.34) | - |  |
| **Diabetes** |  |  |  |  |  |
| Depression | 1.07 (1.07,1.08) | 1.11 (1.10,1.11) | 0.49 (0.49,0.50) | - |  |
| Anxiety | 1.02 (1.02,1.03) | 1.06 (1.06,1.07) | 0.39 (0.39,0.40) | - |  |
| Bulimia nervosa | 1.05 (1.02,1.08) | 1.07 (1.04,1.10) | 0.31 (0.23,0.43) | - |  |
| Other unspecified eating disorders | 1.22 (1.20,1.24) | 1.24 (1.22,1.26) | 0.56 (0.50,0.63) | - |  |
| Bipolar disorders | 1.12 (1.11,1.14) | 1.17 (1.15,1.18) | 0.39 (0.36,0.42) | - |  |
| Schizophrenia | 1.26 (1.24,1.28) | 1.31 (1.29,1.33) | 0.42 (0.39,0.44) | - |  |
| Other psychoses | 1.12 (1.10,1.14) | 1.16 (1.14,1.19) | 0.44 (0.41,0.48) | - |  |
| **Hypertension** |  |  |  |  |  |
| Depression | 1.09 (1.09,1.10) | 1.12 (1.12,1.13) | 0.44 (0.44,0.45) | - |  |
| Anxiety | 1.04 (1.03,1.04) | 1.07 (1.07,1.08) | 0.44 (0.43,0.45) | - |  |
| Bulimia nervosa | 1.04 (1.01,1.07) | 1.06 (1.03,1.10) | 0.24 (0.19,0.29) | - |  |
| Other unspecified eating disorders | 1.22 (1.20,1.24) | 1.25 (1.23,1.27) | 0.43 (0.40,0.47) | - |  |
| Bipolar disorders | 1.12 (1.10,1.14) | 1.17 (1.15,1.18) | 0.30 (0.28,0.31) | - |  |
| Schizophrenia | 1.26 (1.25,1.28) | 1.33 (1.31,1.34) | 0.24 (0.22,0.25) | - |  |
| Other psychoses | 1.13 (1.11,1.15) | 1.18 (1.16,1.21) | 0.33 (0.31,0.36) | - |  |
| Each mediator model and outcome model incorporated age, sex, general practice, ethnicity, socioeconomic status, region; marital status, smoking status, drinking status, physical activity, and comorbidities of chronic obstructive pulmonary disease, obstructive sleep apnoea, renal failure, thyroid disease, hyperglycaemia, anaemia, multiple sclerosis, inflammatory bowel disease, and eczema.  Associations between BMI and mental illness were restricted to individuals with a BMI of ≥21 kg/m^2^. | | | | | |

**Table S2.** Causal mediation analysis to assess the mediation effect of inflammatory and cardiometabolic risk markers for observed associations between BMI and incident mental illness risk.

|  | **Incident depression (N=5,897,835)** | | **Incident anxiety(N=6,568,934)** | | **Incident anorexia nervosa(N=6,877,447)** | | **Incident bulimia nervosa (N=6,881,170)** | | **Incident other unspecified eating disorders(N=6,878,816)** | | **Incident bipolar disorder(N=6,867,769)** | | **Incident schizophrenia(N=6,863,103)** | | **Incident other psychoses (N=6,877,669)** | |
| --- | --- | --- | --- | --- | --- | --- | --- | --- | --- | --- | --- | --- | --- | --- | --- | --- |
|  | **Proportion of mediated associations (95% CI)** | **P-value** | **Proportion of mediated associations (95% CI)** | **P-value** | **Proportion of mediated associations (95% CI)** | **P-value** | **Proportion of mediated associations (95% CI)** | **P-value** | **Proportion of mediated associations (95% CI)** | **P-value** | **Proportion of mediated associations (95% CI)** | **P-value** | **Proportion of mediated associations (95% CI)** | **P-value** | **Proportion of mediated associations (95% CI)** | **P-value** |
| **CRP** | - | - | - | - | - | - | - | - | - | - | - | - | 0.020 (-0.010 to 0.052) | 0.240 | 0.038 (-0.088 to 0.224) | 0.440 |
| **Cholesterol** | - | - | - | - | - | - | - | - | 0.003 (0.002 to 0.005) | <0.001 | - | - | - |  | - | - |
| **LDL** | 0.008 (0.007 to 0.009) | <0.001 | 0.017 (0.015 to 0.019) | <0.001 | 0.000 (-0.001 to 0.002) | 0.580 | - | - | - | - | 0.004 (0.002 to 0.006) | <0.001 | 0.002 (0.001 to 0.004) | <0.001 | 0.004 (0.002 to 0.008) | <0.001 |
| **Triglycerides** | 0.081 (0.077 to 0.087) | <0.001 | 0.109 (0.098 to 0.123) | <0.001 | - | - | - | - | - | - | 0.150 (0.128 to 0.186) | <0.001 | 0.100 (0.090 to 0.112) | <0.001 | 0.122 (0.094 to 0.162) | <0.001 |
| **Systolic** | - | - | - | - | 0.182 (0.157 to 0.207) | <0.001 | - | - | - | - | - | - | - | - | - | - |
| **Diastolic** | - | - | - | - | 0.188 (0.163 to 0.222) | <0.001 | - | - | - | - | - | - | - | - | - | - |
| **HbA1c** | 0.103 (0.099 to 0.108) | <0.001 | - | - | - |  | 0.031 (-0.018 to 0.100) | 0.200 | 0.025 (0.011 to 0.040) | <0.001 | - | - | 0.023 (0.010 to 0.037) | <0.001 | 0.029 (0.002 to 0.049) | 0.020 |

These analyses test the extent to which inflammatory and cardiometabolic risk markers mediate the associations of BMI with incident mental illnesses. Each mediator model and outcome model incorporated age, sex, general practice, ethnicity, socioeconomic status, region; marital status, smoking status, drinking status, physical activity, and comorbidities of chronic obstructive pulmonary disease, obstructive sleep apnoea, renal failure, thyroid disease, hyperglycaemia, anaemia, multiple sclerosis, inflammatory bowel disease, and eczema.

**Table S3.** Adjusted associations between BMI category and mental illness excluding the first 5 years of follow-up after the BMI record in individuals with mental illness

|  | **HR (95%CI) compared with healthy weight** | | | | | |
| --- | --- | --- | --- | --- | --- | --- |
| **Mental illness** | **Underweight**  **(<18.5kg/m^2^)** | **Healthy weight**  **(18.5-25 kg/m^2^)** | **Overweight**  **(25-30 kg/m^2^)** | **Obesity**  **(30-35 kg/m^2^)** | **Obesity**  **(35-40 kg/m^2^)** | **Obesity**  **(≥40 kg/m^2^)** |
| **Depression** |  |  |  |  |  |  |
| Model 1 (unadjusted) | 1.32 (1.29,1.34) | 1.00 (REF) | 0.91 (0.91,0.92) | 1.00 (0.99,1.01) | 1.17 (1.16,1.19) | 1.38 (1.36,1.40) |
| Model 2 (model 1 plus age and sex) | 1.21 (1.19,1.24) | 1.00 (REF) | 1.01 (1.01,1.02) | 1.09 (1.08,1.10) | 1.22 (1.21,1.23) | 1.37 (1.35,1.39) |
| Model 3 (model 2 plus demographic factors) | 1.20 (1.18,1.23) | 1.00 (REF) | 1.01 (1.00,1.01) | 1.07 (1.06,1.08) | 1.17 (1.16,1.18) | 1.29 (1.27,1.31) |
| Model 4 (model 3 plus health behaviours) | 1.19 (1.17,1.21) | 1.00 (REF) | 1.01 (1.00,1.01) | 1.07 (1.06,1.07) | 1.17 (1.16,1.18) | 1.29 (1.27,1.31) |
| Model 5 (model 5 plus obesity-related morbidity) | 1.18 (1.16,1.20) | 1.00 (REF) | 1.00 (1.00,1.01) | 1.04 (1.03,1.05) | 1.13 (1.11,1.14) | 1.23 (1.21,1.25) |
| **Anxiety** |  |  |  |  |  |  |
| Model 1 (unadjusted) | 1.49 (1.46,1.52) | 1.00 (REF) | 0.79 (0.78,0.79) | 0.81 (0.80,0.82) | 0.93 (0.92,0.95) | 1.08 (1.06,1.10) |
| Model 2 (model 1 plus age and sex) | 1.19 (1.17,1.22) | 1.00 (REF) | 0.99 (0.98,0.99) | 1.00 (0.99,1.01) | 1.04 (1.03,1.06) | 1.11 (1.09,1.13) |
| Model 3 (model 2 plus demographic factors) | 1.20 (1.18,1.22) | 1.00 (REF) | 0.98 (0.97,0.99) | 0.98 (0.97,0.99) | 1.01 (0.99,1.02) | 1.05 (1.03,1.07) |
| Model 4 (model 3 plus health behaviours) | 1.19 (1.17,1.21) | 1.00 (REF) | 0.98 (0.97,0.99) | 0.98 (0.97,0.99) | 1.01 (0.99,1.02) | 1.05 (1.03,1.07) |
| Model 5 (model 5 plus obesity-related morbidity) | 1.18 (1.16,1.21) | 1.00 (REF) | 0.98 (0.97,0.99) | 0.98 (0.97,0.99) | 1.00 (0.99,1.02) | 1.05 (1.03,1.07) |
| **Anorexia nervosa** |  |  |  |  |  |  |
| Model 1 (unadjusted) | 2.28 (2.14,2.42) | 1.00 (REF) | 0.88 (0.85,0.90) | 0.86 (0.83,0.90) | 0.77 (0.73,0.82) | 0.70 (0.64,0.77) |
| Model 2 (model 1 plus age and sex) | 2.94 (2.76,3.12) | 1.00 (REF) | 0.74 (0.72,0.76) | 0.76 (0.73,0.78) | 0.74 (0.70,0.79) | 0.76 (0.70,0.83) |
| Model 3 (model 2 plus demographic factors) | 2.71 (2.55,2.88) | 1.00 (REF) | 0.74 (0.71,0.76) | 0.73 (0.71,0.76) | 0.70 (0.66,0.75) | 0.70 (0.64,0.77) |
| Model 4 (model 3 plus health behaviours) | 2.63 (2.48,2.80) | 1.00 (REF) | 0.74 (0.72,0.76) | 0.73 (0.71,0.76) | 0.71 (0.67,0.75) | 0.71 (0.65,0.78) |
| Model 5 (model 5 plus obesity-related morbidity) | 2.61 (2.46,2.77) | 1.00 (REF) | 0.72 (0.70,0.74) | 0.69 (0.66,0.71) | 0.64 (0.60,0.68) | 0.62 (0.57,0.68) |
| **Bulimia nervosa** |  |  |  |  |  |  |
| Model 1 (unadjusted) | 2.35 (1.83,3.01) | 1.00 (REF) | 0.61 (0.53,0.70) | 0.74 (0.63,0.87) | 0.96 (0.77,1.21) | 2.09 (1.66,2.64) |
| Model 2 (model 1 plus age and sex) | 1.32 (1.03,1.70) | 1.00 (REF) | 1.14 (1.00,1.31) | 1.38 (1.17,1.62) | 1.44 (1.15,1.81) | 2.66 (2.11,3.36) |
| Model 3 (model 2 plus demographic factors) | 1.37 (1.07,1.76) | 1.00 (REF) | 1.16 (1.01,1.33) | 1.40 (1.18,1.65) | 1.44 (1.15,1.81) | 2.72 (2.15,3.43) |
| Model 4 (model 3 plus health behaviours) | 1.38 (1.08,1.77) | 1.00 (REF) | 1.15 (1.01,1.32) | 1.39 (1.18,1.64) | 1.44 (1.15,1.81) | 2.73 (2.16,3.45) |
| Model 5 (model 5 plus obesity-related morbidity) | 1.38 (1.07,1.77) | 1.00 (REF) | 1.16 (1.01,1.32) | 1.39 (1.18,1.64) | 1.43 (1.14,1.80) | 2.68 (2.12,3.39) |
| **Other unspecified eating disorders** |  |  |  |  |  |  |
| Model 1 (unadjusted) | 4.89 (4.47,5.35) | 1.00 (REF) | 0.56 (0.52,0.60) | 0.79 (0.73,0.85) | 1.47 (1.34,1.61) | 2.69 (2.43,2.98) |
| Model 2 (model 1 plus age and sex) | 3.11 (2.84,3.41) | 1.00 (REF) | 0.92 (0.86,0.99) | 1.28 (1.18,1.38) | 1.96 (1.79,2.15) | 3.10 (2.80,3.43) |
| Model 3 (model 2 plus demographic factors) | 3.17 (2.89,3.47) | 1.00 (REF) | 0.92 (0.86,0.99) | 1.27 (1.17,1.37) | 1.93 (1.75,2.11) | 3.00 (2.71,3.32) |
| Model 4 (model 3 plus health behaviours) | 3.16 (2.89,3.46) | 1.00 (REF) | 0.92 (0.86,0.99) | 1.26 (1.17,1.36) | 1.92 (1.75,2.10) | 2.99 (2.70,3.31) |
| Model 5 (model 5 plus obesity-related morbidity) | 3.14 (2.87,3.44) | 1.00 (REF) | 0.92 (0.86,0.99) | 1.25 (1.16,1.36) | 1.90 (1.73,2.08) | 2.92 (2.63,3.24) |
| **Bipolar disorders** |  |  |  |  |  |  |
| Model 1 (unadjusted) | 1.28 (1.11,1.48) | 1.00 (REF) | 0.92 (0.88,0.98) | 1.05 (0.98,1.12) | 1.30 (1.19,1.42) | 1.41 (1.24,1.59) |
| Model 2 (model 1 plus age and sex) | 1.08 (0.93,1.25) | 1.00 (REF) | 1.09 (1.04,1.15) | 1.22 (1.15,1.30) | 1.41 (1.29,1.55) | 1.44 (1.27,1.62) |
| Model 3 (model 2 plus demographic factors) | 1.07 (0.92,1.24) | 1.00 (REF) | 1.08 (1.03,1.14) | 1.18 (1.11,1.26) | 1.34 (1.23,1.47) | 1.33 (1.17,1.50) |
| Model 4 (model 3 plus health behaviours) | 1.05 (0.91,1.21) | 1.00 (REF) | 1.09 (1.03,1.15) | 1.19 (1.11,1.27) | 1.35 (1.24,1.48) | 1.34 (1.19,1.52) |
| Model 5 (model 4 plus obesity-related morbidity, depression, and anxiety) | 0.99 (0.86,1.15) | 1.00 (REF) | 1.06 (1.01,1.12) | 1.12 (1.05,1.19) | 1.24 (1.13,1.35) | 1.18 (1.04,1.34) |
| **Schizophrenia** |  |  |  |  |  |  |
| Model 1 (unadjusted) | 1.67 (1.43,1.95) | 1.00 (REF) | 1.03 (0.97,1.10) | 1.37 (1.28,1.47) | 1.70 (1.55,1.87) | 1.84 (1.61,2.09) |
| Model 2 (model 1 plus age and sex) | 1.68 (1.44,1.96) | 1.00 (REF) | 0.98 (0.92,1.05) | 1.32 (1.23,1.42) | 1.70 (1.55,1.87) | 1.90 (1.66,2.16) |
| Model 3 (model 2 plus demographic factors) | 1.51 (1.30,1.76) | 1.00 (REF) | 0.96 (0.91,1.03) | 1.24 (1.16,1.34) | 1.55 (1.41,1.70) | 1.67 (1.47,1.91) |
| Model 4 (model 3 plus health behaviours) | 1.46 (1.25,1.70) | 1.00 (REF) | 0.99 (0.93,1.05) | 1.27 (1.19,1.37) | 1.59 (1.44,1.75) | 1.71 (1.50,1.95) |
| Model 5 (model 4 plus obesity-related morbidity, depression, and anxiety) | 1.43 (1.23,1.67) | 1.00 (REF) | 0.98 (0.92,1.05) | 1.25 (1.17,1.35) | 1.54 (1.40,1.69) | 1.62 (1.42,1.85) |
| **Other psychoses** |  |  |  |  |  |  |
| Model 1 (unadjusted) | 1.36 (1.12,1.65) | 1.00 (REF) | 1.04 (0.97,1.11) | 1.22 (1.12,1.33) | 1.45 (1.29,1.63) | 1.56 (1.33,1.83) |
| Model 2 (model 1 plus age and sex) | 1.34 (1.10,1.62) | 1.00 (REF) | 1.05 (0.98,1.13) | 1.23 (1.13,1.34) | 1.46 (1.30,1.64) | 1.56 (1.33,1.83) |
| Model 3 (model 2 plus demographic factors) | 1.29 (1.07,1.57) | 1.00 (REF) | 1.03 (0.95,1.10) | 1.16 (1.06,1.26) | 1.34 (1.19,1.50) | 1.39 (1.19,1.64) |
| Model 4 (model 3 plus health behaviours) | 1.26 (1.04,1.53) | 1.00 (REF) | 1.03 (0.96,1.11) | 1.17 (1.07,1.27) | 1.35 (1.20,1.51) | 1.40 (1.19,1.65) |
| Model 5 (model 4 plus obesity-related morbidity, depression, and anxiety) | 1.23 (1.01,1.48) | 1.00 (REF) | 1.02 (0.95,1.10) | 1.13 (1.04,1.23) | 1.28 (1.13,1.43) | 1.29 (1.10,1.52) |
| Model 5 adjusted for age, sex, general practitioner, ethnicity, socioeconomic status, region, marital status, smoking status, drinking status, physical activity, cardiovascular disease, type 2 diabetes, hypertension, chronic obstructive pulmonary disease, obstructive sleep apnoea, renal failure, thyroid disease, hyperglycaemia, anaemia, multiple sclerosis, inflammatory bowel disease, and eczema. | | | | | | |

**Table S4.** Adjusted associations between BMI category and mental illness after excluding individuals over 40 years old at baseline

|  | **HR (95%CI) compared with healthy weight** | | | | | |
| --- | --- | --- | --- | --- | --- | --- |
| **Mental illness** | **Underweight**  **(<18.5kg/m^2^)** | **Healthy weight**  **(18.5-25 kg/m^2^)** | **Overweight**  **(25-30 kg/m^2^)** | **Obesity**  **(30-35 kg/m^2^)** | **Obesity**  **(35-40 kg/m^2^)** | **Obesity**  **(≥40 kg/m^2^)** |
| **Depression** |  |  |  |  |  |  |
| Model 1 (unadjusted) | 1.31 (1.30,1.32) | 1.00 (REF) | 0.99 (0.98,0.99) | 1.16 (1.15,1.17) | 1.36 (1.35,1.38) | 1.62 (1.60,1.64) |
| Model 2 (model 1 plus age and sex) | 1.22 (1.21,1.24) | 1.00 (REF) | 1.09 (1.09,1.10) | 1.28 (1.27,1.29) | 1.47 (1.45,1.48) | 1.70 (1.68,1.72) |
| Model 3 (model 2 plus demographic factors) | 1.26 (1.24,1.27) | 1.00 (REF) | 1.08 (1.08,1.09) | 1.23 (1.23,1.24) | 1.37 (1.36,1.39) | 1.55 (1.53,1.57) |
| Model 4 (model 3 plus health behaviours) | 1.25 (1.23,1.26) | 1.00 (REF) | 1.08 (1.07,1.08) | 1.22 (1.21,1.23) | 1.36 (1.35,1.38) | 1.54 (1.52,1.56) |
| Model 5 (model 5 plus obesity-related morbidity) | 1.25 (1.23,1.26) | 1.00 (REF) | 1.08 (1.07,1.08) | 1.22 (1.21,1.23) | 1.35 (1.34,1.36) | 1.52 (1.50,1.54) |
| **Anxiety** |  |  |  |  |  |  |
| Model 1 (unadjusted) | 1.36 (1.34,1.38) | 1.00 (REF) | 0.92 (0.91,0.92) | 1.01 (1.00,1.02) | 1.14 (1.13,1.16) | 1.30 (1.28,1.32) |
| Model 2 (model 1 plus age and sex) | 1.23 (1.21,1.25) | 1.00 (REF) | 1.03 (1.02,1.03) | 1.13 (1.12,1.14) | 1.24 (1.23,1.26) | 1.39 (1.37,1.41) |
| Model 3 (model 2 plus demographic factors) | 1.26 (1.24,1.28) | 1.00 (REF) | 1.02 (1.01,1.03) | 1.10 (1.09,1.11) | 1.18 (1.17,1.19) | 1.29 (1.27,1.31) |
| Model 4 (model 3 plus health behaviours) | 1.25 (1.24,1.27) | 1.00 (REF) | 1.01 (1.01,1.02) | 1.10 (1.09,1.11) | 1.17 (1.16,1.19) | 1.29 (1.27,1.31) |
| Model 5 (model 5 plus obesity-related morbidity) | 1.25 (1.24,1.27) | 1.00 (REF) | 1.01 (1.01,1.02) | 1.09 (1.08,1.10) | 1.17 (1.15,1.18) | 1.27 (1.25,1.29) |
| **Anorexia nervosa** |  |  |  |  |  |  |
| Model 1 (unadjusted) | 5.33 (5.10,5.56) | 1.00 (REF) | 0.54 (0.52,0.57) | 0.55 (0.51,0.59) | 0.54 (0.49,0.60) | 0.54 (0.47,0.62) |
| Model 2 (model 1 plus age and sex) | 4.92 (4.71,5.14) | 1.00 (REF) | 0.61 (0.58,0.63) | 0.61 (0.57,0.65) | 0.58 (0.53,0.65) | 0.57 (0.49,0.65) |
| Model 3 (model 2 plus demographic factors) | 4.92 (4.71,5.14) | 1.00 (REF) | 0.59 (0.57,0.62) | 0.58 (0.54,0.62) | 0.54 (0.49,0.60) | 0.51 (0.45,0.59) |
| Model 4 (model 3 plus health behaviours) | 4.85 (4.64,5.07) | 1.00 (REF) | 0.59 (0.57,0.62) | 0.58 (0.54,0.62) | 0.54 (0.49,0.60) | 0.51 (0.45,0.59) |
| Model 5 (model 5 plus obesity-related morbidity) | 4.83 (4.62,5.04) | 1.00 (REF) | 0.59 (0.56,0.62) | 0.57 (0.53,0.61) | 0.53 (0.48,0.58) | 0.49 (0.43,0.56) |
| **Bulimia nervosa** |  |  |  |  |  |  |
| Model 1 (unadjusted) | 1.62 (1.46,1.80) | 1.00 (REF) | 0.65 (0.61,0.70) | 0.65 (0.59,0.71) | 0.79 (0.70,0.90) | 1.13 (0.97,1.31) |
| Model 2 (model 1 plus age and sex) | 1.35 (1.22,1.51) | 1.00 (REF) | 0.85 (0.79,0.91) | 0.83 (0.76,0.91) | 0.94 (0.83,1.07) | 1.29 (1.11,1.49) |
| Model 3 (model 2 plus demographic factors) | 1.41 (1.27,1.56) | 1.00 (REF) | 0.87 (0.81,0.93) | 0.86 (0.78,0.94) | 0.97 (0.85,1.10) | 1.31 (1.13,1.53) |
| Model 4 (model 3 plus health behaviours) | 1.41 (1.27,1.57) | 1.00 (REF) | 0.86 (0.80,0.92) | 0.85 (0.78,0.94) | 0.97 (0.85,1.10) | 1.32 (1.13,1.53) |
| Model 5 (model 5 plus obesity-related morbidity) | 1.41 (1.27,1.57) | 1.00 (REF) | 0.86 (0.80,0.92) | 0.85 (0.77,0.93) | 0.95 (0.84,1.09) | 1.28 (1.10,1.49) |
| **Other unspecified eating disorders** |  |  |  |  |  |  |
| Model 1 (unadjusted) | 4.83 (4.64,5.02) | 1.00 (REF) | 0.55 (0.53,0.57) | 0.67 (0.63,0.71) | 1.03 (0.96,1.10) | 1.71 (1.59,1.84) |
| Model 2 (model 1 plus age and sex) | 3.93 (3.78,4.09) | 1.00 (REF) | 0.71 (0.68,0.74) | 0.87 (0.82,0.91) | 1.25 (1.17,1.34) | 2.01 (1.87,2.16) |
| Model 3 (model 2 plus demographic factors) | 4.08 (3.92,4.25) | 1.00 (REF) | 0.72 (0.69,0.75) | 0.88 (0.83,0.93) | 1.25 (1.17,1.34) | 1.99 (1.85,2.14) |
| Model 4 (model 3 plus health behaviours) | 4.11 (3.95,4.28) | 1.00 (REF) | 0.72 (0.69,0.75) | 0.88 (0.83,0.93) | 1.25 (1.17,1.34) | 1.99 (1.85,2.14) |
| Model 5 (model 5 plus obesity-related morbidity) | 4.09 (3.93,4.26) | 1.00 (REF) | 0.72 (0.69,0.75) | 0.87 (0.83,0.92) | 1.24 (1.16,1.32) | 1.94 (1.80,2.09) |
| **Bipolar disorders** |  |  |  |  |  |  |
| Model 1 (unadjusted) | 1.08 (0.97,1.19) | 1.00 (REF) | 1.19 (1.14,1.24) | 1.48 (1.40,1.56) | 1.80 (1.68,1.94) | 2.02 (1.84,2.21) |
| Model 2 (model 1 plus age and sex) | 1.10 (1.00,1.22) | 1.00 (REF) | 1.19 (1.14,1.25) | 1.48 (1.40,1.56) | 1.79 (1.67,1.92) | 1.98 (1.81,2.17) |
| Model 3 (model 2 plus demographic factors) | 1.11 (1.00,1.22) | 1.00 (REF) | 1.18 (1.13,1.23) | 1.42 (1.35,1.50) | 1.67 (1.56,1.79) | 1.79 (1.63,1.96) |
| Model 4 (model 3 plus health behaviours) | 1.09 (0.98,1.20) | 1.00 (REF) | 1.18 (1.12,1.23) | 1.41 (1.34,1.49) | 1.66 (1.55,1.78) | 1.79 (1.64,1.96) |
| Model 5 (model 4 plus obesity-related morbidity, depression, and anxiety) | 0.99 (0.89,1.09) | 1.00 (REF) | 1.15 (1.10,1.20) | 1.31 (1.24,1.38) | 1.46 (1.36,1.57) | 1.49 (1.35,1.63) |
| **Schizophrenia** |  |  |  |  |  |  |
| Model 1 (unadjusted) | 1.43 (1.29,1.58) | 1.00 (REF) | 1.42 (1.35,1.50) | 2.13 (2.02,2.26) | 2.49 (2.31,2.68) | 2.79 (2.54,3.06) |
| Model 2 (model 1 plus age and sex) | 1.61 (1.45,1.79) | 1.00 (REF) | 1.11 (1.05,1.17) | 1.70 (1.61,1.80) | 2.19 (2.03,2.36) | 2.67 (2.43,2.93) |
| Model 3 (model 2 plus demographic factors) | 1.52 (1.37,1.68) | 1.00 (REF) | 1.09 (1.04,1.15) | 1.63 (1.54,1.72) | 2.07 (1.92,2.23) | 2.46 (2.24,2.70) |
| Model 4 (model 3 plus health behaviours) | 1.45 (1.31,1.61) | 1.00 (REF) | 1.12 (1.07,1.18) | 1.67 (1.57,1.76) | 2.11 (1.96,2.27) | 2.52 (2.29,2.76) |
| Model 5 (model 4 plus obesity-related morbidity, depression, and anxiety) | 1.38 (1.25,1.53) | 1.00 (REF) | 1.12 (1.07,1.18) | 1.63 (1.54,1.72) | 2.00 (1.85,2.15) | 2.28 (2.08,2.51) |
| **Other psychoses** |  |  |  |  |  |  |
| Model 1 (unadjusted) | 1.21 (1.05,1.40) | 1.00 (REF) | 1.18 (1.10,1.26) | 1.60 (1.48,1.73) | 1.75 (1.57,1.95) | 1.77 (1.53,2.04) |
| Model 2 (model 1 plus age and sex) | 1.29 (1.12,1.49) | 1.00 (REF) | 1.05 (0.98,1.12) | 1.44 (1.33,1.56) | 1.64 (1.48,1.83) | 1.72 (1.49,1.98) |
| Model 3 (model 2 plus demographic factors) | 1.25 (1.09,1.44) | 1.00 (REF) | 1.02 (0.95,1.09) | 1.35 (1.25,1.46) | 1.51 (1.36,1.68) | 1.56 (1.35,1.79) |
| Model 4 (model 3 plus health behaviours) | 1.22 (1.06,1.40) | 1.00 (REF) | 1.03 (0.96,1.10) | 1.36 (1.26,1.47) | 1.52 (1.36,1.69) | 1.57 (1.36,1.81) |
| Model 5 (model 4 plus obesity-related morbidity, depression, and anxiety) | 1.14 (0.99,1.31) | 1.00 (REF) | 1.02 (0.96,1.09) | 1.30 (1.20,1.41) | 1.40 (1.26,1.56) | 1.38 (1.20,1.60) |
| Model 5 adjusted for age, sex, general practitioner, ethnicity, socioeconomic status, region, marital status, smoking status, drinking status, physical activity, cardiovascular disease, type 2 diabetes, hypertension, chronic obstructive pulmonary disease, obstructive sleep apnoea, renal failure, thyroid disease, hyperglycaemia, anaemia, multiple sclerosis, inflammatory bowel disease, and eczema. | | | | | | |

**Table S5.** Adjusted associations between BMI category and mental illness excluding individuals with cardiovascular disease, hypertension, and type 2 diabetes at baseline

|  | **HR (95%CI) compared with healthy weight** | | | | | |
| --- | --- | --- | --- | --- | --- | --- |
| **Mental illness** | **Underweight**  **(<18.5kg/m^2^)** | **Healthy weight**  **(18.5-25 kg/m^2^)** | **Overweight**  **(25-30 kg/m^2^)** | **Obesity**  **(30-35 kg/m^2^)** | **Obesity**  **(35-40 kg/m^2^)** | **Obesity**  **(≥40 kg/m^2^)** |
| **Depression** |  |  |  |  |  |  |
| Model 1 (unadjusted) | 1.44 (1.43,1.46) | 1.00 (REF) | 0.89 (0.88,0.89) | 1.02 (1.02,1.03) | 1.25 (1.24,1.26) | 1.53 (1.51,1.55) |
| Model 2 (model 1 plus age and sex) | 1.29 (1.28,1.30) | 1.00 (REF) | 1.03 (1.03,1.04) | 1.16 (1.16,1.17) | 1.33 (1.32,1.34) | 1.53 (1.51,1.54) |
| Model 3 (model 2 plus demographic factors) | 1.29 (1.28,1.30) | 1.00 (REF) | 1.03 (1.02,1.03) | 1.13 (1.13,1.14) | 1.26 (1.25,1.27) | 1.42 (1.40,1.43) |
| Model 4 (model 3 plus health behaviours) | 1.28 (1.27,1.29) | 1.00 (REF) | 1.03 (1.02,1.03) | 1.13 (1.12,1.13) | 1.25 (1.24,1.26) | 1.41 (1.40,1.43) |
| Model 5 (model 5 plus obesity-related morbidity) | 1.27 (1.26,1.28) | 1.00 (REF) | 1.03 (1.02,1.03) | 1.13 (1.12,1.13) | 1.25 (1.24,1.26) | 1.41 (1.40,1.43) |
| **Anxiety** |  |  |  |  |  |  |
| Model 1 (unadjusted) | 1.49 (1.47,1.51) | 1.00 (REF) | 0.92 (0.91,0.92) | 0.90 (0.90,0.91) | 1.07 (1.06,1.08) | 1.26 (1.24,1.28) |
| Model 2 (model 1 plus age and sex) | 1.26 (1.25,1.28) | 1.00 (REF) | 0.99 (0.99,1.00) | 1.06 (1.05,1.07) | 1.14 (1.13,1.15) | 1.25 (1.24,1.27) |
| Model 3 (model 2 plus demographic factors) | 1.28 (1.26,1.29) | 1.00 (REF) | 0.99 (0.98,0.99) | 1.03 (1.03,1.04) | 1.09 (1.08,1.10) | 1.18 (1.16,1.19) |
| Model 4 (model 3 plus health behaviours) | 1.27 (1.25,1.28) | 1.00 (REF) | 0.98 (0.98,0.99) | 1.03 (1.02,1.04) | 1.09 (1.08,1.10) | 1.17 (1.16,1.19) |
| Model 5 (model 5 plus obesity-related morbidity) | 1.26 (1.25,1.28) | 1.00 (REF) | 0.98 (0.98,0.99) | 1.03 (1.02,1.04) | 1.09 (1.07,1.10) | 1.17 (1.15,1.19) |
| **Anorexia nervosa** |  |  |  |  |  |  |
| Model 1 (unadjusted) | 4.73 (4.57,4.89) | 1.00 (REF) | 0.62 (0.61,0.64) | 0.58 (0.55,0.60) | 0.54 (0.50,0.57) | 0.50 (0.45,0.56) |
| Model 2 (model 1 plus age and sex) | 5.17 (4.99,5.35) | 1.00 (REF) | 0.59 (0.58,0.61) | 0.56 (0.53,0.58) | 0.53 (0.50,0.57) | 0.51 (0.46,0.56) |
| Model 3 (model 2 plus demographic factors) | 4.80 (4.64,4.96) | 1.00 (REF) | 0.59 (0.58,0.61) | 0.54 (0.52,0.56) | 0.50 (0.47,0.53) | 0.46 (0.42,0.51) |
| Model 4 (model 3 plus health behaviours) | 4.66 (4.50,4.82) | 1.00 (REF) | 0.60 (0.58,0.61) | 0.54 (0.52,0.56) | 0.50 (0.47,0.53) | 0.46 (0.42,0.51) |
| Model 5 (model 5 plus obesity-related morbidity) | 4.51 (4.36,4.67) | 1.00 (REF) | 0.60 (0.58,0.62) | 0.54 (0.52,0.57) | 0.50 (0.47,0.54) | 0.47 (0.42,0.52) |
| **Bulimia nervosa** |  |  |  |  |  |  |
| Model 1 (unadjusted) | 2.12 (1.92,2.35) | 1.00 (REF) | 0.50 (0.47,0.53) | 0.54 (0.50,0.59) | 0.78 (0.69,0.89) | 1.13 (0.97,1.32) |
| Model 2 (model 1 plus age and sex) | 1.43 (1.29,1.58) | 1.00 (REF) | 0.83 (0.77,0.88) | 0.85 (0.78,0.93) | 0.99 (0.88,1.12) | 1.24 (1.07,1.45) |
| Model 3 (model 2 plus demographic factors) | 1.48 (1.34,1.64) | 1.00 (REF) | 0.84 (0.79,0.90) | 0.87 (0.80,0.96) | 1.03 (0.92,1.17) | 1.27 (1.09,1.48) |
| Model 4 (model 3 plus health behaviours) | 1.50 (1.35,1.66) | 1.00 (REF) | 0.84 (0.79,0.90) | 0.87 (0.80,0.95) | 1.03 (0.91,1.17) | 1.27 (1.09,1.48) |
| Model 5 (model 5 plus obesity-related morbidity) | 1.50 (1.35,1.66) | 1.00 (REF) | 0.84 (0.78,0.90) | 0.87 (0.79,0.95) | 1.03 (0.91,1.16) | 1.26 (1.08,1.46) |
| **Other unspecified eating disorders** |  |  |  |  |  |  |
| Model 1 (unadjusted) | 6.23 (6.00,6.46) | 1.00 (REF) | 0.43 (0.41,0.45) | 0.57 (0.54,0.60) | 1.03 (0.97,1.10) | 1.91 (1.78,2.04) |
| Model 2 (model 1 plus age and sex) | 4.31 (4.15,4.47) | 1.00 (REF) | 0.68 (0.65,0.71) | 0.86 (0.82,0.91) | 1.28 (1.21,1.36) | 2.07 (1.93,2.21) |
| Model 3 (model 2 plus demographic factors) | 4.45 (4.28,4.62) | 1.00 (REF) | 0.69 (0.66,0.71) | 0.87 (0.83,0.92) | 1.28 (1.20,1.36) | 2.03 (1.90,2.17) |
| Model 4 (model 3 plus health behaviours) | 4.47 (4.31,4.65) | 1.00 (REF) | 0.68 (0.66,0.71) | 0.87 (0.83,0.91) | 1.28 (1.20,1.36) | 2.03 (1.89,2.17) |
| Model 5 (model 5 plus obesity-related morbidity) | 4.46 (4.29,4.63) | 1.00 (REF) | 0.68 (0.66,0.71) | 0.87 (0.83,0.91) | 1.27 (1.19,1.35) | 2.01 (1.88,2.15) |
| **Bipolar disorders** |  |  |  |  |  |  |
| Model 1 (unadjusted) | 1.18 (1.09,1.29) | 1.00 (REF) | 1.06 (1.03,1.10) | 1.35 (1.30,1.41) | 1.70 (1.61,1.80) | 1.92 (1.78,2.07) |
| Model 2 (model 1 plus age and sex) | 1.12 (1.03,1.22) | 1.00 (REF) | 1.15 (1.11,1.19) | 1.44 (1.38,1.50) | 1.73 (1.64,1.83) | 1.90 (1.76,2.05) |
| Model 3 (model 2 plus demographic factors) | 1.11 (1.02,1.20) | 1.00 (REF) | 1.14 (1.10,1.17) | 1.38 (1.33,1.44) | 1.62 (1.53,1.71) | 1.72 (1.59,1.86) |
| Model 4 (model 3 plus health behaviours) | 1.09 (1.00,1.19) | 1.00 (REF) | 1.14 (1.10,1.17) | 1.38 (1.32,1.43) | 1.61 (1.53,1.71) | 1.72 (1.60,1.86) |
| Model 5 (model 4 plus obesity-related morbidity, depression, and anxiety) | 1.02 (0.94,1.11) | 1.00 (REF) | 1.10 (1.06,1.14) | 1.27 (1.22,1.32) | 1.43 (1.35,1.51) | 1.46 (1.35,1.57) |
| **Schizophrenia** |  |  |  |  |  |  |
| Model 1 (unadjusted) | 1.68 (1.56,1.81) | 1.00 (REF) | 1.13 (1.09,1.17) | 1.62 (1.56,1.69) | 2.06 (1.95,2.17) | 2.39 (2.23,2.58) |
| Model 2 (model 1 plus age and sex) | 1.79 (1.66,1.93) | 1.00 (REF) | 0.98 (0.94,1.01) | 1.45 (1.39,1.51) | 2.00 (1.89,2.12) | 2.51 (2.34,2.71) |
| Model 3 (model 2 plus demographic factors) | 1.63 (1.51,1.76) | 1.00 (REF) | 0.97 (0.94,1.00) | 1.38 (1.32,1.44) | 1.86 (1.76,1.96) | 2.24 (2.08,2.41) |
| Model 4 (model 3 plus health behaviours) | 1.56 (1.45,1.68) | 1.00 (REF) | 1.00 (0.96,1.03) | 1.41 (1.36,1.47) | 1.89 (1.79,2.00) | 2.28 (2.12,2.46) |
| Model 5 (model 4 plus obesity-related morbidity, depression, and anxiety) | 1.51 (1.39,1.62) | 1.00 (REF) | 0.99 (0.96,1.03) | 1.38 (1.33,1.44) | 1.82 (1.73,1.93) | 2.16 (2.01,2.33) |
| **Other psychoses** |  |  |  |  |  |  |
| Model 1 (unadjusted) | 1.42 (1.27,1.58) | 1.00 (REF) | 1.07 (1.02,1.12) | 1.37 (1.30,1.45) | 1.58 (1.46,1.72) | 1.73 (1.55,1.94) |
| Model 2 (model 1 plus age and sex) | 1.43 (1.28,1.60) | 1.00 (REF) | 1.03 (0.98,1.08) | 1.33 (1.26,1.41) | 1.57 (1.45,1.71) | 1.75 (1.57,1.96) |
| Model 3 (model 2 plus demographic factors) | 1.37 (1.23,1.53) | 1.00 (REF) | 1.01 (0.96,1.06) | 1.26 (1.19,1.33) | 1.44 (1.33,1.56) | 1.57 (1.40,1.75) |
| Model 4 (model 3 plus health behaviours) | 1.34 (1.20,1.49) | 1.00 (REF) | 1.02 (0.97,1.07) | 1.26 (1.19,1.34) | 1.45 (1.34,1.57) | 1.57 (1.41,1.76) |
| Model 5 (model 4 plus obesity-related morbidity, depression, and anxiety) | 1.28 (1.15,1.43) | 1.00 (REF) | 1.00 (0.96,1.05) | 1.21 (1.14,1.28) | 1.35 (1.25,1.47) | 1.44 (1.28,1.61) |
| Model 5 adjusted for age, sex, general practitioner, ethnicity, socioeconomic status, region, marital status, smoking status, drinking status, physical activity, cardiovascular disease, type 2 diabetes, hypertension, chronic obstructive pulmonary disease, obstructive sleep apnoea, renal failure, thyroid disease, hyperglycaemia, anaemia, multiple sclerosis, inflammatory bowel disease, and eczema. | | | | | | |

**Table S6.** Adjusted associations between BMI category and mental illness in individuals who had a BMI record less than 1 year after registration

|  | **HR (95%CI) compared with healthy weight** | | | | | |
| --- | --- | --- | --- | --- | --- | --- |
| **Mental illness** | **Underweight**  **(<18.5kg/m^2^)** | **Healthy weight**  **(18.5-25 kg/m^2^)** | **Overweight**  **(25-30 kg/m^2^)** | **Obesity**  **(30-35 kg/m^2^)** | **Obesity**  **(35-40 kg/m^2^)** | **Obesity**  **(≥40 kg/m^2^)** |
| **Depression** |  |  |  |  |  |  |
| Model 1 (unadjusted) | 1.40 (1.39,1.42) | 1.00 (REF) | 0.94 (0.93,0.94) | 1.06 (1.05,1.06) | 1.26 (1.25,1.28) | 1.54 (1.52,1.56) |
| Model 2 (model 1 plus age and sex) | 1.32 (1.30,1.34) | 1.00 (REF) | 1.06 (1.06,1.07) | 1.20 (1.20,1.21) | 1.39 (1.37,1.40) | 1.63 (1.61,1.65) |
| Model 3 (model 2 plus demographic factors) | 1.31 (1.30,1.33) | 1.00 (REF) | 1.06 (1.06,1.07) | 1.19 (1.18,1.20) | 1.34 (1.32,1.35) | 1.54 (1.52,1.56) |
| Model 4 (model 3 plus health behaviours) | 1.30 (1.28,1.31) | 1.00 (REF) | 1.06 (1.05,1.07) | 1.18 (1.17,1.19) | 1.32 (1.31,1.34) | 1.52 (1.50,1.54) |
| Model 5 (model 5 plus obesity-related morbidity) | 1.28 (1.27,1.30) | 1.00 (REF) | 1.06 (1.06,1.07) | 1.17 (1.16,1.18) | 1.30 (1.29,1.32) | 1.48 (1.47,1.50) |
| **Anxiety** |  |  |  |  |  |  |
| Model 1 (unadjusted) | 1.48 (1.45,1.50) | 1.00 (REF) | 0.81 (0.81,0.82) | 0.86 (0.86,0.87) | 1.01 (1.00,1.02) | 1.19 (1.17,1.21) |
| Model 2 (model 1 plus age and sex) | 1.29 (1.27,1.31) | 1.00 (REF) | 1.02 (1.01,1.03) | 1.11 (1.10,1.12) | 1.22 (1.21,1.24) | 1.37 (1.35,1.39) |
| Model 3 (model 2 plus demographic factors) | 1.30 (1.28,1.32) | 1.00 (REF) | 1.02 (1.01,1.03) | 1.09 (1.08,1.10) | 1.18 (1.16,1.19) | 1.28 (1.26,1.30) |
| Model 4 (model 3 plus health behaviours) | 1.29 (1.27,1.31) | 1.00 (REF) | 1.02 (1.01,1.03) | 1.08 (1.07,1.09) | 1.17 (1.15,1.18) | 1.27 (1.25,1.29) |
| Model 5 (model 5 plus obesity-related morbidity) | 1.28 (1.26,1.30) | 1.00 (REF) | 1.02 (1.01,1.03) | 1.08 (1.07,1.10) | 1.16 (1.15,1.18) | 1.26 (1.24,1.28) |
| **Anorexia nervosa** |  |  |  |  |  |  |
| Model 1 (unadjusted) | 4.18 (3.99,4.37) | 1.00 (REF) | 0.79 (0.76,0.82) | 0.72 (0.69,0.76) | 0.65 (0.61,0.70) | 0.55 (0.49,0.61) |
| Model 2 (model 1 plus age and sex) | 4.96 (4.74,5.19) | 1.00 (REF) | 0.65 (0.62,0.67) | 0.59 (0.56,0.62) | 0.56 (0.52,0.59) | 0.49 (0.44,0.55) |
| Model 3 (model 2 plus demographic factors) | 4.66 (4.45,4.88) | 1.00 (REF) | 0.65 (0.63,0.67) | 0.58 (0.56,0.61) | 0.54 (0.50,0.58) | 0.47 (0.43,0.52) |
| Model 4 (model 3 plus health behaviours) | 4.52 (4.31,4.73) | 1.00 (REF) | 0.65 (0.63,0.68) | 0.58 (0.56,0.61) | 0.54 (0.50,0.58) | 0.47 (0.42,0.52) |
| Model 5 (model 5 plus obesity-related morbidity) | 4.46 (4.26,4.67) | 1.00 (REF) | 0.64 (0.62,0.66) | 0.55 (0.52,0.57) | 0.49 (0.46,0.52) | 0.42 (0.38,0.47) |
| **Bulimia nervosa** |  |  |  |  |  |  |
| Model 1 (unadjusted) | 1.93 (1.69,2.20) | 1.00 (REF) | 0.47 (0.43,0.52) | 0.45 (0.40,0.51) | 0.64 (0.55,0.74) | 0.80 (0.66,0.97) |
| Model 2 (model 1 plus age and sex) | 1.41 (1.24,1.61) | 1.00 (REF) | 0.85 (0.78,0.93) | 0.90 (0.80,1.02) | 1.15 (0.98,1.34) | 1.32 (1.09,1.60) |
| Model 3 (model 2 plus demographic factors) | 1.46 (1.28,1.66) | 1.00 (REF) | 0.87 (0.80,0.95) | 0.94 (0.83,1.06) | 1.19 (1.02,1.39) | 1.38 (1.14,1.66) |
| Model 4 (model 3 plus health behaviours) | 1.47 (1.28,1.67) | 1.00 (REF) | 0.86 (0.79,0.95) | 0.93 (0.83,1.05) | 1.18 (1.01,1.37) | 1.37 (1.13,1.65) |
| Model 5 (model 5 plus obesity-related morbidity) | 1.47 (1.28,1.67) | 1.00 (REF) | 0.87 (0.79,0.95) | 0.93 (0.83,1.05) | 1.17 (1.01,1.37) | 1.35 (1.11,1.63) |
| **Other unspecified eating disorders** |  |  |  |  |  |  |
| Model 1 (unadjusted) | 5.66 (5.39,5.95) | 1.00 (REF) | 0.40 (0.38,0.42) | 0.43 (0.41,0.47) | 0.72 (0.66,0.78) | 1.37 (1.26,1.49) |
| Model 2 (model 1 plus age and sex) | 4.23 (4.03,4.45) | 1.00 (REF) | 0.68 (0.65,0.72) | 0.82 (0.77,0.88) | 1.24 (1.14,1.34) | 2.15 (1.98,2.34) |
| Model 3 (model 2 plus demographic factors) | 4.35 (4.14,4.57) | 1.00 (REF) | 0.69 (0.65,0.73) | 0.84 (0.78,0.89) | 1.24 (1.14,1.35) | 2.12 (1.95,2.31) |
| Model 4 (model 3 plus health behaviours) | 4.36 (4.14,4.58) | 1.00 (REF) | 0.69 (0.65,0.73) | 0.83 (0.78,0.89) | 1.23 (1.14,1.34) | 2.11 (1.94,2.30) |
| Model 5 (model 5 plus obesity-related morbidity) | 4.33 (4.12,4.55) | 1.00 (REF) | 0.69 (0.65,0.73) | 0.82 (0.77,0.88) | 1.21 (1.11,1.31) | 2.02 (1.85,2.20) |
| **Bipolar disorders** |  |  |  |  |  |  |
| Model 1 (unadjusted) | 1.11 (0.99,1.24) | 1.00 (REF) | 1.23 (1.18,1.29) | 1.53 (1.46,1.61) | 1.92 (1.80,2.05) | 2.20 (2.03,2.38) |
| Model 2 (model 1 plus age and sex) | 1.08 (0.96,1.21) | 1.00 (REF) | 1.29 (1.23,1.35) | 1.61 (1.53,1.70) | 2.00 (1.87,2.13) | 2.26 (2.08,2.45) |
| Model 3 (model 2 plus demographic factors) | 1.08 (0.96,1.21) | 1.00 (REF) | 1.27 (1.21,1.33) | 1.54 (1.46,1.62) | 1.85 (1.74,1.98) | 2.02 (1.86,2.19) |
| Model 4 (model 3 plus health behaviours) | 1.06 (0.95,1.19) | 1.00 (REF) | 1.26 (1.21,1.32) | 1.52 (1.44,1.60) | 1.82 (1.70,1.94) | 1.98 (1.82,2.15) |
| Model 5 (model 4 plus obesity-related morbidity, depression, and anxiety) | 0.97 (0.87,1.09) | 1.00 (REF) | 1.23 (1.18,1.29) | 1.42 (1.35,1.50) | 1.60 (1.50,1.71) | 1.62 (1.49,1.76) |
| **Schizophrenia** |  |  |  |  |  |  |
| Model 1 (unadjusted) | 1.40 (1.27,1.55) | 1.00 (REF) | 1.36 (1.30,1.43) | 1.95 (1.86,2.04) | 2.41 (2.27,2.56) | 2.68 (2.49,2.88) |
| Model 2 (model 1 plus age and sex) | 1.49 (1.35,1.65) | 1.00 (REF) | 1.14 (1.09,1.20) | 1.65 (1.57,1.73) | 2.20 (2.07,2.34) | 2.62 (2.44,2.83) |
| Model 3 (model 2 plus demographic factors) | 1.43 (1.29,1.58) | 1.00 (REF) | 1.11 (1.06,1.16) | 1.54 (1.47,1.62) | 2.00 (1.88,2.12) | 2.31 (2.14,2.49) |
| Model 4 (model 3 plus health behaviours) | 1.36 (1.23,1.51) | 1.00 (REF) | 1.14 (1.09,1.19) | 1.58 (1.51,1.66) | 2.03 (1.91,2.15) | 2.32 (2.16,2.51) |
| Model 5 (model 4 plus obesity-related morbidity, depression, and anxiety) | 1.30 (1.18,1.44) | 1.00 (REF) | 1.15 (1.10,1.20) | 1.60 (1.53,1.68) | 2.02 (1.91,2.15) | 2.24 (2.08,2.42) |
| **Other psychoses** |  |  |  |  |  |  |
| Model 1 (unadjusted) | 1.19 (1.01,1.40) | 1.00 (REF) | 1.18 (1.11,1.26) | 1.54 (1.43,1.66) | 1.90 (1.73,2.09) | 2.05 (1.82,2.32) |
| Model 2 (model 1 plus age and sex) | 1.22 (1.04,1.43) | 1.00 (REF) | 1.11 (1.03,1.18) | 1.45 (1.35,1.56) | 1.84 (1.67,2.02) | 2.03 (1.80,2.29) |
| Model 3 (model 2 plus demographic factors) | 1.20 (1.03,1.41) | 1.00 (REF) | 1.07 (1.00,1.14) | 1.34 (1.25,1.45) | 1.64 (1.50,1.81) | 1.78 (1.58,2.01) |
| Model 4 (model 3 plus health behaviours) | 1.17 (1.00,1.37) | 1.00 (REF) | 1.08 (1.01,1.15) | 1.35 (1.25,1.45) | 1.64 (1.49,1.80) | 1.77 (1.57,2.00) |
| Model 5 (model 4 plus obesity-related morbidity, depression, and anxiety) | 1.10 (0.94,1.29) | 1.00 (REF) | 1.07 (1.00,1.14) | 1.32 (1.22,1.42) | 1.55 (1.41,1.71) | 1.60 (1.42,1.81) |
| Model 5 adjusted for age, sex, general practitioner, ethnicity, socioeconomic status, region, marital status, smoking status, drinking status, physical activity, cardiovascular disease, type 2 diabetes, hypertension, chronic obstructive pulmonary disease, obstructive sleep apnoea, renal failure, thyroid disease, hyperglycaemia, anaemia, multiple sclerosis, inflammatory bowel disease, and eczema. | | | | | | |

**Table S7.** Adjusted associations between BMI category and SMI using the diagnosis date of SMI as the incident date

|  | **HR (95%CI) compared with healthy weight** | | | | | |
| --- | --- | --- | --- | --- | --- | --- |
| **Mental illness** | **Underweight**  **(<18.5kg/m^2^)** | **Healthy weight**  **(18.5-25 kg/m^2^)** | **Overweight**  **(25-30 kg/m^2^)** | **Obesity**  **(30-35 kg/m^2^)** | **Obesity**  **(35-40 kg/m^2^)** | **Obesity**  **(≥40 kg/m^2^)** |
| **Bipolar disorders** |  |  |  |  |  |  |
| Model 1 (unadjusted) | 1.19 (1.10,1.28) | 1.00 (REF) | 1.03 (1.00,1.06) | 1.26 (1.22,1.31) | 1.65 (1.58,1.73) | 1.86 (1.75,1.98) |
| Model 2 (model 1 plus age and sex) | 1.08 (1.00,1.17) | 1.00 (REF) | 1.15 (1.11,1.18) | 1.40 (1.35,1.45) | 1.75 (1.67,1.83) | 1.89 (1.78,2.01) |
| Model 3 (model 2 plus demographic factors) | 1.07 (0.99,1.16) | 1.00 (REF) | 1.14 (1.10,1.17) | 1.34 (1.30,1.39) | 1.64 (1.56,1.71) | 1.72 (1.62,1.83) |
| Model 4 (model 3 plus health behaviours) | 1.05 (0.97,1.14) | 1.00 (REF) | 1.14 (1.10,1.17) | 1.34 (1.29,1.39) | 1.63 (1.56,1.71) | 1.72 (1.61,1.82) |
| Model 5 (model 4 plus obesity-related morbidity, depression, and anxiety) | 0.99 (0.92,1.07) | 1.00 (REF) | 1.10 (1.07,1.14) | 1.24 (1.20,1.28) | 1.44 (1.37,1.51) | 1.42 (1.34,1.51) |
| **Schizophrenia** |  |  |  |  |  |  |
| Model 1 (unadjusted) | 1.63 (1.52,1.75) | 1.00 (REF) | 1.10 (1.07,1.13) | 1.53 (1.48,1.58) | 1.99 (1.90,2.07) | 2.29 (2.17,2.43) |
| Model 2 (model 1 plus age and sex) | 1.66 (1.55,1.78) | 1.00 (REF) | 1.01 (0.98,1.04) | 1.44 (1.39,1.49) | 1.98 (1.89,2.06) | 2.39 (2.26,2.53) |
| Model 3 (model 2 plus demographic factors) | 1.53 (1.43,1.64) | 1.00 (REF) | 1.00 (0.97,1.03) | 1.35 (1.31,1.40) | 1.80 (1.73,1.88) | 2.11 (1.99,2.23) |
| Model 4 (model 3 plus health behaviours) | 1.47 (1.37,1.57) | 1.00 (REF) | 1.02 (0.99,1.05) | 1.39 (1.34,1.43) | 1.84 (1.76,1.92) | 2.14 (2.02,2.27) |
| Model 5 (model 4 plus obesity-related morbidity, depression, and anxiety) | 1.42 (1.33,1.52) | 1.00 (REF) | 1.02 (0.99,1.05) | 1.36 (1.31,1.40) | 1.75 (1.67,1.82) | 1.96 (1.85,2.07) |
| **Other psychoses** |  |  |  |  |  |  |
| Model 1 (unadjusted) | 1.35 (1.22,1.49) | 1.00 (REF) | 1.04 (1.00,1.08) | 1.26 (1.21,1.32) | 1.52 (1.43,1.62) | 1.70 (1.56,1.84) |
| Model 2 (model 1 plus age and sex) | 1.31 (1.19,1.45) | 1.00 (REF) | 1.05 (1.01,1.09) | 1.27 (1.21,1.33) | 1.54 (1.45,1.64) | 1.72 (1.58,1.87) |
| Model 3 (model 2 plus demographic factors) | 1.26 (1.14,1.40) | 1.00 (REF) | 1.02 (0.98,1.06) | 1.19 (1.14,1.25) | 1.41 (1.32,1.50) | 1.53 (1.41,1.67) |
| Model 4 (model 3 plus health behaviours) | 1.23 (1.12,1.36) | 1.00 (REF) | 1.03 (0.99,1.07) | 1.20 (1.15,1.26) | 1.41 (1.33,1.51) | 1.54 (1.42,1.68) |
| Model 5 (model 4 plus obesity-related morbidity, depression, and anxiety) | 1.18 (1.07,1.31) | 1.00 (REF) | 1.02 (0.98,1.06) | 1.15 (1.10,1.21) | 1.32 (1.24,1.41) | 1.39 (1.27,1.51) |
| Model 5 adjusted for age, sex, general practitioner, ethnicity, socioeconomic status, region, marital status, smoking status, drinking status, physical activity, cardiovascular disease, type 2 diabetes, hypertension, chronic obstructive pulmonary disease, obstructive sleep apnoea, renal failure, thyroid disease, hyperglycaemia, anaemia, multiple sclerosis, inflammatory bowel disease, and eczema. | | | | | | |

**Table S8.** Adjusted associations between BMI category and SMI excluding individuals with common mental illness at baseline

|  | **HR (95%CI) compared with healthy weight** | | | | | |
| --- | --- | --- | --- | --- | --- | --- |
| **Mental illness** | **Underweight**  **(<18.5kg/m^2^)** | **Healthy weight**  **(18.5-25 kg/m^2^)** | **Overweight**  **(25-30 kg/m^2^)** | **Obesity**  **(30-35 kg/m^2^)** | **Obesity**  **(35-40 kg/m^2^)** | **Obesity**  **(≥40 kg/m^2^)** |
| **Bipolar disorders** | 1.12 (1.01,1.25) | 1.00 (REF) | 1.09 (1.05,1.14) | 1.26 (1.20,1.32) | 1.53 (1.43,1.63) | 1.60 (1.46,1.75) |
| **Schizophrenia** | 1.51 (1.38,1.64) | 1.00 (REF) | 0.97 (0.93,1.00) | 1.32 (1.27,1.38) | 1.74 (1.65,1.84) | 2.02 (1.88,2.17) |
| **Other psychoses** | 1.36 (1.19,1.54) | 1.00 (REF) | 0.96 (0.91,1.02) | 1.15 (1.08,1.22) | 1.40 (1.29,1.52) | 1.39 (1.24,1.57) |
| The model was adjusted for age, sex, general practice, ethnicity, socioeconomic status, region; marital status, smoking status, drinking status, physical activity, and comorbidities of CVD, diabetes, hypertension, chronic obstructive pulmonary disease, obstructive sleep apnoea, renal failure, thyroid disease, hyperglycaemia, anaemia, multiple sclerosis, inflammatory bowel disease, skin and soft tissue infection, eczema, back pain, osteoarthritis, and gout. | | | | | | |

**Table S9.** Adjusted associations between BMI category and mental illness after imputation

|  | **HR (95%CI) compared with healthy weight** | | | | | |
| --- | --- | --- | --- | --- | --- | --- |
| **Mental illness** | **Underweight**  **(<18.5kg/m^2^)** | **Healthy weight**  **(18.5-25 kg/m^2^)** | **Overweight**  **(25-30 kg/m^2^)** | **Obesity**  **(30-35 kg/m^2^)** | **Obesity**  **(35-40 kg/m^2^)** | **Obesity**  **(≥40 kg/m^2^)** |
| **Depression** | 1.30 (1.28,1.31) | 1.00 (REF) | 1.01 (1.00,1.02) | 1.09 (1.08,1.09) | 1.20 (1.19,1.21) | 1.35 (1.33,1.36) |
| **Anxiety** | 1.26 (1.25, 1.28) | 1.00 (REF) | 0.95 (0.97, 0.98) | 1.01 (1.00, 1.01) | 1.06 (1.05, 1.06) | 1.14 (1.12, 1.14) |
| **Anorexia nervosa** | 4.01 (3.93, 4.18) | 1.00 (REF) | 0.63 (0.62, 0.64) | 0.56 (0.54, 0.57) | 0.51 (0.48, 0.53) | 0.45 (0.42, 0.49) |
| **Bulimia nervosa** | 1.50 (1.36,1.67) | 1.00 (REF) | 0.81 (0.71,0.83) | 0.84 (0.78,0.93) | 1.02 (0.91,1.14) | 1.30 (1.14,1.53) |
| **Other unspecified eating disorders** | 4.48 (4.32,4.67) | 1.00 (REF) | 0.66 (0.64,0.70) | 0.83 (0.79,0.87) | 1.20 (1.13,1.29) | 1.98 (1.87,2.10) |
| **Bipolar disorders** | 1.03 (1.01,1.10) | 1.00 (REF) | 1.12 (1.11,1.17) | 1.24 (1.22,1.43) | 1.49 (1.40,1.57) | 1.48 (1.46,1.60) |
| **Schizophrenia** | 1.46 (1.33,1.60) | 1.00 (REF) | 1.02 (1.01,1.06) | 1.40 (1.34,1.46) | 1.76 (1.66,1.84) | 2.03 (2.00,2.18) |
| **Other psychoses** | 1.23 (1.15,1.43) | 1.00 (REF) | 1.03 (1.00,1.08) | 1.20 (1.11,1.34) | 1..37 (1.34,1.56) | 1.42 (1.31,1.62) |
| The model was adjusted for age, sex, general practice, ethnicity, socioeconomic status, region; marital status, smoking status, drinking status, physical activity, and comorbidities of CVD, diabetes, hypertension, chronic obstructive pulmonary disease, obstructive sleep apnoea, renal failure, thyroid disease, hyperglycaemia, anaemia, multiple sclerosis, inflammatory bowel disease, skin and soft tissue infection, eczema, back pain, osteoarthritis, and gout. | | | | | | |
